# Biologic Therapy for Inflammatory Bowel Disease: Real-World Comparative Effectiveness and Impact of Drug Sequencing in 13 222 Patients within the UK IBD BioResource

**DOI:** 10.1093/ecco-jcc/jjad203

**Published:** 2023-12-02

**Authors:** Christina Kapizioni, Rofaida Desoki, Danielle Lam, Karthiha Balendran, Eman Al-Sulais, Sreedhar Subramanian, Joanna E Rimmer, Juan De La Revilla Negro, Holly Pavey, Laetitia Pele, Johanne Brooks, Gordon W Moran, Peter M Irving, Jimmy K Limdi, Christopher A Lamb, UK IBD BioResource Investigators, Christopher Alexakis, Mohammed Allah-Ditta, Richard Appleby, Bijay Baburajan, Michelle Baker-Moffatt, Tyara Banerjee, Paul Banim, John Beckly, Roisin Bevan, Stuart Bloom, Monica Bose, Elaine Brinkworth, Johanne Brooks, Deborah Butcher, Jeffrey Butterworth, Monica Chan, Katie Clark, Andrew Cole, Joseph Collum, Rachel Cooney, Fraser Cummings, Albert Davies, Aminda De Silva, John DeCaestecker, Anjan Dhar, Stacey Duffy, Dharmaraj Durai, Cathryn Edwards, Stephen Foley, Tessa Glazebrook, John Gordon, Michael Grimes, Anton Gunasekera, Laura Hancock, Mina Hanna, Ailsa Hart, Gini Hay, David Hobday, Patricia Hooper, Mark Jarvis, Babur Javaid, Matthew Johnson, Lijo Joy, Rzwan Kassam, Nick Kennedy, Alexandra Kent, Klaartje Bel Kok, Konrad Koss, Nicola Lancaster, Jonathan Landy, Charlie Lees, Wendy Lewis, Stephen Lewis, Andy Li, Alan Lobo, Juliette Loehry, Chris Macdonald, Christopher Macdonald, George Macfaul, Zahid Mahmood, Dina Mansour, Simon McLaughlin, John McLaughlin, Yin Miao, Ajay Muddu, Charles Murray, Chuka Nwokolo, Susan O’Sullivan, Abby Oglesby, Simon Panter, Vinod Patel, Linda Patterson, Ruth Penn, Anne Phillips, Kath Phillis, Richard Pollok, Sam Powles, Cathryn Preston, Monira Rahman, Arvind Ramadas, John Ramage, Subramaniam Ramakrishnan, Jack Satsangi, John Saunders, Glyn Scott, Shali Sebastian, Christian Selinger, Sherif Shabana, Rakesh Shah, Dan Sharpstone, Sophy Shedwell, Christopher Sheen, Richard Shenderey, Achuth Shenoy, Alison Simmons, Salil Singh, Leena Sinha, Ganesh Sivaji, Melissa Smith, Paul Smith, Katherine Smith, Helen Steed, Alan Steel, Byron Theron, Jude Tidbury, Theresa Tindall, Mark Tremelling, Deven Vani, Ajay Verma, Gareth Walker, Ben Warner, Alastair Watson, Emma Wesley, Alan Wiles, Joy Wilkins, Horace Williams, Miles Parkes, Tim Raine

**Affiliations:** Department of Gastroenterology, Cambridge University Hospitals NHS Foundation Trust, Cambridge, UK; Department of Gastroenterology, Attikon University Hospital, Athens, Greece; Department of Gastroenterology, Cambridge University Hospitals NHS Foundation Trust, Cambridge, UK; Department of Genetics, Faculty of Medicine, Alexandria University, Alexandria, Egypt; Department of Gastroenterology, Cambridge University Hospitals NHS Foundation Trust, Cambridge, UK; Department of Gastroenterology, Sir Charles Gairdner Hospital, Perth, Australia; Department of Gastroenterology, Cambridge University Hospitals NHS Foundation Trust, Cambridge, UK; Department of Clinical Medicine, University of Jaffna, Sri Lanka; Department of Gastroenterology, Cambridge University Hospitals NHS Foundation Trust, Cambridge, UK; King Fahad Specialist Hospital, Dammam, Saudi Arabia; Department of Gastroenterology, Cambridge University Hospitals NHS Foundation Trust, Cambridge, UK; Academic Department of Military Medicine, Royal Centre for Defence Medicine, Medical Directorate, Joint Medical Command, Birmingham Research Park, Birmingham, UK; Department of Gastroenterology, Cambridge University Hospitals NHS Foundation Trust, Cambridge, UK; Division of Experimental Medicine and Immunotherapeutics, Department of Medicine, University of Cambridge, Cambridge, UK; Institute of Health Economics, Medical University Innsbruck, Innsbruck, Austria; Department of Medicine, University of Cambridge, Cambridge, UK; IBD BioResource, NIHR BioResource, Cambridge, UK; Department of Clinical Pharmacology and Biological Sciences, University of Hertfordshire, Hatfield, UK; Gastroenterology Department, Lister Hospital, Stevenage, UK; University of Nottingham, NIHR Nottingham Biomedical Research Centre, Nottingham, UK; Department of Gastroenterology, Guy’s and St Thomas’ NHS Foundation Trust, London, UK; School of Immunology and Microbial Sciences, King’s College London, London, UK; IBD Section – Northern Care Alliance NHS Foundation Trust, Manchester, UK; Faculty of Biology, Medicine & Health, University of Manchester, Manchester, UK; Clinical and Translational Research Institute, Newcastle University, Newcastle upon Tyne, UK; Department of Gastroenterology, Newcastle upon Tyne Hospitals NHS Foundation Trust, Newcastle upon Tyne, UK; Department of Gastroenterology, Cambridge University Hospitals NHS Foundation Trust, Cambridge, UK; Department of Gastroenterology, Cambridge University Hospitals NHS Foundation Trust, Cambridge, UK

**Keywords:** Crohn’s disease, ulcerative colitis, biologic therapy, sequencing, real-world effectiveness

## Abstract

**Background and Aims:**

This study compares the effectiveness of different biologic therapies and sequences in patients with inflammatory bowel disease [IBD] using real-world data from a large cohort with long exposure.

**Methods:**

Demographic, disease, treatment, and outcome data were retrieved for patients in the UK IBD BioResource. Effectiveness of treatment was based on persistence free of discontinuation or failure, analysed by Kaplan–Meier survival analysis with inverse probability of treatment weighting to adjust for differences between groups.

**Results:**

In total, 13 222 evaluable patients received at least one biologic. In ulcerative colitis [UC] first-line vedolizumab [VDZ] demonstrated superior effectiveness over 5 years compared to anti-tumour necrosis factor [anti-TNF] agents [*p* = 0.006]. VDZ was superior to both infliximab [IFX] and adalimumab [ADA] after ADA and IFX failure respectively [*p* < 0.001 and *p* < 0.001]. Anti-TNF therapy showed similar effectiveness when used as first-line treatment, or after failure of VDZ. In Crohn’s disease [CD] we found significant differences between first-line treatments over 10 years [*p* = 0.045], with superior effectiveness of IFX compared to ADA in perianal CD. Non-anti-TNF biologics were superior to a second anti-TNF after first-line anti-TNF failure in CD [*p* = 0.035]. Patients with UC or CD experiencing TNF failure due to delayed loss of response or intolerance had superior outcomes when switching to a non-anti-TNF biologic, rather than a second anti-TNF.

**Conclusions:**

We provide real-world evidence to guide biologic selection and sequencing in a range of common scenarios. Our findings challenge current guidelines regarding drug selection after loss of response to first anti-TNF treatment.

## 1. Introduction

Crohn’s disease [CD] and ulcerative colitis [UC] are chronic relapsing–remitting forms of inflammatory bowel disease [IBD].^1^ Since the 1990s anti-tumour necrosis factor [anti-TNF] agents have dramatically improved outcomes, particularly in moderate/severe IBD.^2,3^ Nonetheless, many patients do not respond, or lose response to anti-TNFs, while others are intolerant or experience side effects. In the last decade, more biologic agents and small molecules with different mechanisms of action have been licensed for the treatment of both UC and CD, offering options for both first-line and subsequent therapies. However, there are limited data on comparative efficacy^4,5^ and considerable data gaps regarding drug efficacy when used in different sequential order.

Data from large clinical cohorts can provide valuable insight into drug performance when used in ‘real-world’ settings, including for patients not eligible for clinical trials.^6–8^ A critical challenge is to assemble sufficiently large cohorts to allow meaningful comparisons between groups, and to capture baseline differences between groups and adjust outcomes for these. Inverse probability of treatment weighting [IPTW] is a propensity score method used to balance baseline patient characteristics in groups with different exposures. This can be used to adjust for confounding in observational studies and support inferences about relative outcomes with different interventions.

Here we aimed to assess the comparative effectiveness of biologic therapies in a national cohort of well-characterized IBD patients registered in the UK IBD BioResource using IPTW to assess outcomes associated with the use of different biologics, including different sequences of drugs and in different subpopulations.

## 2. Materials and Methods

### 2.1. Study population

The study included patients diagnosed with CD or UC based on conventional criteria by local physicians, who were registered in the UK IBD BioResource and who initiated treatment with at least one biologic (infliximab [IFX], adalimumab [ADA], golimumab [GLM, only for UC patients], vedolizumab [VDZ], ustekinumab [UST, only for CD patients due to licence restrictions during most of the enrolment period]). Patients without a confirmed IBD diagnosis or with IBD-unspecified were excluded. Biologics were used in line with standard clinical practice and standard dosing regimens, including dose-escalation at the treating physician’s discretion.

All participants provided written informed consent. Participants were recruited from 106 hospitals, mostly between January 2017 and January 2020. At data extraction on January 4, 2022, 36 126 patients had been enrolled into the IBD BioResource, including 16 826 diagnosed with CD and 16 103 with UC. IBD phenotype data, including drug therapy start/stop dates, outcomes, and surgical interventions, were ascertained at enrolment by research nurses and clinicians using a combination of case note review, patient interview, and patient questionnaire. Data for drug exposure prior to enrolment [including any biologic use before 2017] were acquired retrospectively, with prospective updating at subsequent clinical encounters. Periodic data validation exercises were undertaken with independent reassessment of phenotype data. Data validity was secured by built-in control and validation tests, manual data standardization, and random audits of case ascertainment and data quality.^9^

### 2.2. Statistical analysis

The primary outcome was drug effectiveness, based on treatment persistence free of treatment discontinuation or treatment failure. Failure was defined as either the occurrence of resectional or defunctioning bowel surgery while on treatment, or clinician coding of treatment failure. Coding of treatment outcomes was required for all recorded biologics: clinicians were required to select from a list of options which included outcomes indicating success [clinical remission, clinical response short of remission] or treatment failure. Treatment failure codes enabled further sub-classification as primary non-response [PNR] or non-primary non-response [NPNR] covering all other modes of treatment failure [drug intolerance, side effects and secondary loss of response]. In rare cases where coding was ambiguous or missing, we assigned cases as PNR or NPNR if time on the drug was less than or more than 12 months respectively. Neither drug dose optimization/escalation nor perianal surgery for perianal CD were taken as indicating treatment failure where patients stayed on the drug.

For each patient/drug event, follow-up began at the recorded biologic start date and continued until treatment discontinuation. Patients without a recorded discontinuation date were censored at the earliest point of: [a] last known follow-up, [b] date of any resectional or defunctioning bowel surgery, or [c] using date of their recorded clinician assessment codes if indicative of when treatment response was lost. We used median imputation in instances of partially missing dates [e.g. a year recorded but missing details of day/month] or where a stop date was not captured but where mode of failure was recorded. Treatments captured, but with an absent start date, or where a stop date was absent and the time point of censoring could not be estimated, were excluded from analysis.

Continuous parametric variables were reported as mean and standard deviation [SD] and compared using Student’s t-test. Non-parametric variables were reported as median [with interquartile range] and groups compared using a Wilcoxon rank sum test [two group comparisons] or a Kruskal–Wallis rank sum test [three or more group comparisons]. Categorical variables were expressed as frequencies and compared using a chi-squared test.

We used IPTW to adjust for baseline imbalance between groups. Baseline variables which were considered as potentially predictive of treatment outcome and incorporated into the IPTW analysis were sex, disease duration, and age at the start of treatment under consideration, use of combination therapy with immunomodulators, presence of extra-intestinal manifestations, reason for discontinuation of biologic immediately prior to the current treatment [PNR vs NPNR where appropriate], and use of intravenous steroids at the start of each treatment. Also in CD we included smoking history, involvement of the upper gastrointestinal tract, and previous intestinal resection or perianal intervention. The Montreal disease classification was used for additional baseline covariates in the IPTW analysis: disease extent [UC], disease distribution [CD], disease behaviour [CD], and the presence of perianal disease [CD]. Patients were classed as having perianal disease if they developed perianal involvement at any point, including prior to or after starting therapy. Perianal disease included patients with any history of physician-documented perianal CD, or any history of CD-related perianal surgery. Missing data were imputed using the R MICE package.^10^

Balance before and after IPTW was assessed using the standardized mean difference [SMD] between groups. An SMD > 0.1 is usually considered to indicate significant imbalance.^11^ Kaplan–Meier curves were plotted for survival free of treatment discontinuation or failure. Individual curves were truncated where numbers at risk fell below 20. Plots were truncated at the first integer year after only a single remaining group had 20 or more patients at risk. The differences of distributions between medications were compared using the log-rank test. Cox proportional hazards regression analysis was used to compare outcomes between specific treatments. A value of *p* < 0.05 was considered significant.

Statistical analysis was performed using R software version 4.0.3 [10-10-2020].

## 3. Results

### 3.1. Biologic therapy usage within the cohort

After data cleaning, a total of 13 222 evaluable patients exposed to at least one biologic therapy were analysed [4185 UC; 9037 CD]. In total, 9529 patients had exposure to a single biologic, 2876 to two biologics, and 694/119/4 had exposure to 3/4/5 biologics respectively.

**Figure 1.**
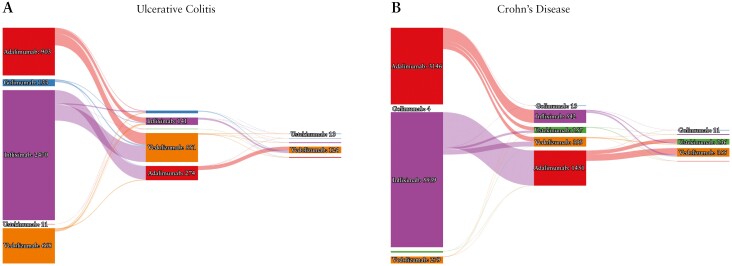
Sequence and frequency of biologic selection for patients with Crohn’s disease [CD] and ulcerative colitis [UC] in the UK IBD BioResource. Sankey diagrams represent frequency of selection in different lines of therapy in patients with UC [A] and CD [B].

Choice of biologic and treatment sequencing is summarized in Figure 1. IFX was the dominant first-line therapy [2470 UC; 5539 CD]. In total, 3506 patients with UC and 8689 patients with CD received an anti-TNF therapy as first-line treatment. In total, 1031 patients with UC and 2662 patients with CD received a second-line biologic therapy. Anti-TNF therapies were again dominant in this position in CD but not in UC, with 469 [45%] and 2006 [75%] patients with UC and CD respectively receiving a second-line anti-TNF therapy. Data on third-line therapy were available for 170 patients with UC and 647 patients with CD.

### 3.2. Differential effectiveness of first-line advanced therapies

#### 3.2.1. Ulcerative colitis

**Figure 2.**
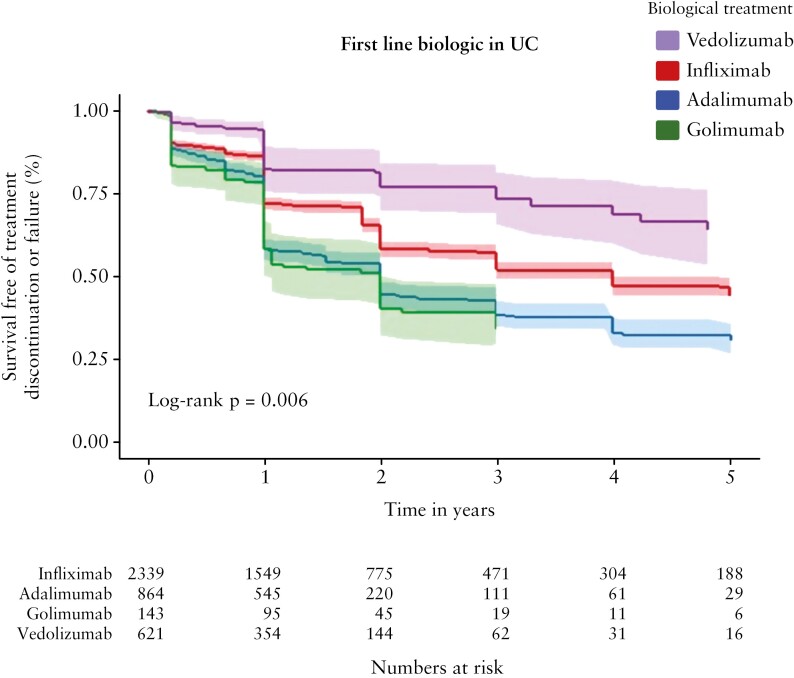
Effectiveness of first-line biologics in patients with ulcerative colitis [UC]. Kaplan–Meier plots depict survival free of treatment discontinuation or failure after inverse probability of treatment weighting adjustment for patients with UC treated for up to 5 years with different biologics as first-line therapy. Log-rank *p*-value as shown.

Analysable data for accurate determination of treatment outcomes were available for 3967 patients with UC who started a first-line biologic therapy. The baseline characteristics of the patients are shown in Supplementary Table 1. Patients receiving first-line VDZ had a higher median age compared to IFX, ADA, and GLM [48 vs 37, 40, and 41 years old respectively]. IPTW adjustment for baseline covariates was successful based upon SMD minimization [Supplementary Figure 1]. Over 5 years of follow-up, treatment survival free of discontinuation or failure was significantly different between treatment groups [Figure 2, *p* = 0.006]. Using a Cox proportional hazards model, we found that VDZ was superior to anti-TNF agents. With VDZ as the reference drug, hazard ratios [HRs] and 95% confidence intervals [CIs] for treatment failure or discontinuation were 3.4 [2.2–5.2] for GLM, 3.1 [2.1–4.5] for ADA, and 1.9 [1.3–2.8] for IFX [all *p* < 0.001]. In direct comparison between anti-TNF agents, IFX had superior survival free of treatment discontinuation or failure compared to ADA and GLM [survival at 5 years: IFX 41%, ADA 25%, GLM 24%]. A proportional hazards model with IFX as the reference drug showed HRs and 95% CIs for treatment failure or discontinuation were 1.6 [1.4–1.8] for ADA and 1.7 [1.3–2.2] for GLM [both *p* < 0.001]. Similar comparisons between ADA and GLM did not show significant differences in outcomes.

#### 3.2.2. Crohn’s disease

Evaluable data were available for first-line therapy in 8780 patients treated with anti-TNF or VDZ. This cohort was sufficient to enable follow-up for up to 10 years [anti-TNF] and 3 years [VDZ]. Patient baseline characteristics are shown in Supplementary Table 2. As in UC, the median age was higher in patients who received first-line VDZ [57 years old] compared to anti-TNF agents [32 and 35 years old for IFX and ADA respectively] [*p* < 0.001]. Most notable were baseline differences in the proportion with perianal disease treated with the different agents, with clear preference given to first-line IFX in the population with perianal disease. IPTW controlled well for most baseline differences, but the SMD after weighting for the presence of perianal disease showed suboptimal correction for this variable [Supplementary Figure 2]. Since only 69 evaluable participants started first-line UST, we excluded them from further analysis. Likewise, we excluded GLM from all analyses in CD.

Rates [and 95% CIs] of survival free of treatment discontinuation or failure for IFX at 1, 3, 5 and 10 years respectively were 75.6% [74.3–76.7%], 54.5% [53.0–56.1%], 44.9% [43.3–46.6%], and 34.5% [32.6–36.5%]. For ADA equivalent rates were 74.3% [72.6–76.0%], 49.8% [47.7–52.1%], 39.2% [36.8–41.7%], and 24.1% [20.9–27.8%]. For VDZ rates at 1 and 3 years were 73.8% [62.5–87.2%] and 69.5% [57.5–84.0%].

**Figure 3.**
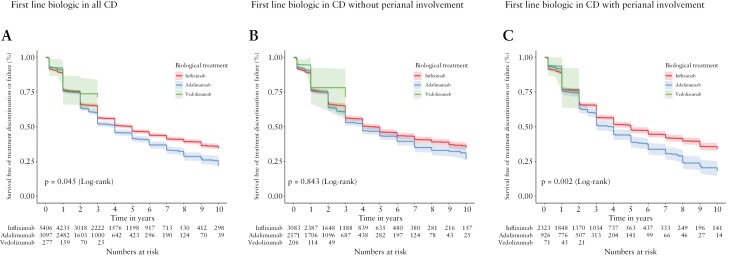
Effectiveness of first line biologics in patients with Crohn’s disease [CD]. Kaplan–Meier plots depict survival free of treatment discontinuation or failure after inverse probability of treatment weighting adjustment for patients with CD treated for up to 10 years with different biologics as first-line therapy. [A] All patients with CD receiving the indicated biologic therapy. [B] Subset of patients with CD and absence of perianal disease. [C] Subset of patients with CD and presence of perianal disease. Log-rank *p* values as shown.

The difference in effectiveness of each agent as first-line treatment in CD was statistically significant [*p* = 0.045], with an apparent advantage for IFX over ADA therapies over 10 years of follow-up [Figure 3A]. Due to imperfect balancing after IPTW for perianal disease, we performed separate subgroup testing using populations of patients with and without perianal CD [3320 and 5460 evaluable participants respectively, Supplementary Tables 3 and 4]. Within these subpopulations, IPTW achieved satisfactory SMD values across all remaining variables. Differences between treatments were not apparent when considering just the subset of patients without perianal CD [Figure 3B]. For the subpopulation CD with perianal disease, we found that IFX was superior to ADA but not VDZ (vs ADA HR 1.26 [95% CI 1.13–1.40], vs VDZ HR 0.82 [95% CI 0.40–1.67]) while comparing VDZ and ADA there was no significant difference (vs ADA HR 1.54 [95% CI 0.75–3.18]) [Figure 3C], although comparisons to VDZ were limited to 3 years of follow-up and by small patient numbers.

### 3.3. Second-line agents after anti-TNF failure

#### 3.3.1. Ulcerative colitis

We next sought to understand drug effectiveness of second-line biologics used in the commonly encountered clinical context of prior anti-TNF failure. In UC, due to the difference in the apparent effectiveness of different anti-TNF drugs, we sought to analyse effectiveness after ADA and IFX failure separately. We did not analyse the effectiveness of second-line biologics after GLM failure due to low patient numbers.

**Figure 4.**
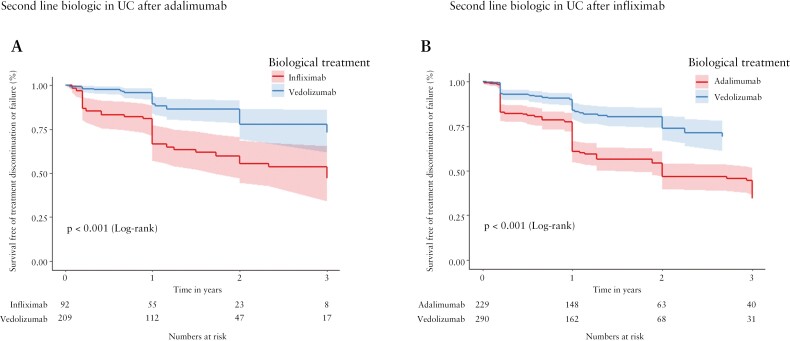
Effectiveness of second-line biologics in patients with ulcerative colitis [UC] after failure of first-line anti-tumour necrosis factor. Kaplan–Meier plots depict survival free of treatment discontinuation or failure after inverse probability of treatment weighting adjustment for patients with UC treated for up to 3 years. [A] Patients treated with infliximab [red] or vedolizumab [blue] after failure of adalimumab [log-rank *p* < 0.001]. [B] Patients treated with adalimumab [red] or vedolizumab [blue] after failure of infliximab [log-rank *p* < 0.001].

In total, 301 evaluable patients with UC who received ADA as their first biologic agent went on to receive either IFX [*n* = 92] or VDZ [*n* = 209] after ADA treatment failure [Supplementary Table 5 and Supplementary Figure 3]. Second-line VDZ demonstrated superior outcomes up to the 3-year follow-up, compared to second-line IFX therapy [Figure 4A, *p* < 0.001].

In total, 229 and 290 evaluable patients with UC received second-line ADA and VDZ respectively, after IFX treatment failure [Supplementary Table 6 and Supplementary Figure 4]. IPTW Kaplan–Meier analysis showed that second-line VDZ was superior to second-line ADA therapy [Figure 4B, *p* < 0.001].

We next sought to understand whether the mode of failure of first-line anti-TNF influenced the comparative effectiveness of second-line biologics. With PNR to IFX, we observed significantly better treatment survival free of discontinuation or failure for those treated with VDZ compared to ADA up to 2 years of follow-up [Supplementary Figure 5A, *p* < 0.001]. In contrast, in PNR to ADA, we found no significant differences between those who received second-line VDZ or IFX [Supplementary Figure 5B], although patient numbers were small.

For NPNR to IFX, VDZ therapy had significantly better effectiveness than ADA [Supplementary Figure 5C, *p* < 0.001], with similar findings for superiority of second-line VDZ compared to IFX after NPNR to ADA [Supplementary Figure 5D, *p* < 0.001].

#### 3.3.2. Crohn’s disease

For analysis of second-line therapy after anti-TNF failure in CD, we first explored whether there were differences in outcome according to selection of a non-anti-TNF vs an anti-TNF agent.

**Figure 5.**
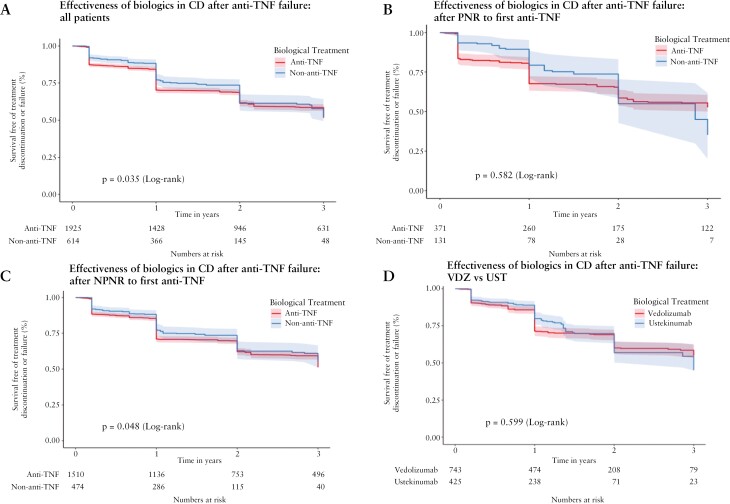
Effectiveness of therapy in Crohn’s disease [CD] after failure of first-line anti-tumour necrosis factor [anti-TNF]. Kaplan–Meier plots depict survival free of treatment discontinuation or failure after inverse probability of treatment weighting adjustment for patients with CD treated for up to 3 years. [A–C] Use of a second anti-TNF [red] compared to a non-anti-TNF biologic [blue] as second-line therapy after failure of a first-line anti-TNF: [A] in all eligible patients with CD, [B] in patients who experienced primary non-response to first-line anti-TNF, and [C] in patients who experienced non-primary non-response to first-line anti-TNF. [D] Comparison of patients treated with vedlizumab [red] and ustekinumab [blue] as second- or third-line therapy after failure of prior anti-TNF therapy. Log-rank *p* values as shown.

In total, 2539 evaluable patients with CD who received first-line anti-TNF therapy subsequently received either second-line anti-TNF [*n* = 1925] or a non-anti-TNF biologic [UST or VDZ, *n* = 614] [Supplementary Table 7]. Importantly, good correction for imbalances in the presence of perianal disease and all other covariates was achieved after IPTW [Supplementary Figure 6]. Patients treated with non-anti-TNF agents showed evidence of superior treatment effectiveness compared to a second-line anti-TNF, although the absolute differences were small and mostly reflected differences in the first 12–24 months of therapy [Figure 5A].

Again, we analysed these findings according to the mode of failure of the first-line anti-TNF. After PNR to a first anti-TNF [*n* = 502], we found no difference between second-line anti-TNF vs non-anti-TNF biologics after IPTW [Figure 5B, *p* = 0.582]. However, in the case of NPNR to a first anti-TNF [*n* = 1984], non-anti-TNF biologics appeared slightly more effective compared to anti-TNF biologics. This persisted after IPTW [Figure 5C, *p* = 0.048].

Another common clinical choice is whether to use UST or VDZ following anti-TNF treatment failure. We identified 1168 evaluable patients with anti-TNF refractory CD who received either VDZ or UST as either second-line or third-line treatment after two prior anti-TNF therapies [Supplementary Table 8 and Supplementary Figure 7a]. IPTW Kaplan–Meier analysis demonstrated similar effectiveness between both groups over 3 years of follow-up [*p* = 0.599] [Figure 5D].

Although disease location was a baseline covariate for IPTW, we also tested subgroup analyses stratified according to disease location [Supplementary Figures 7B–-D and 8A–C] as well as history of perianal disease [Supplementary Figures 7E and 8D]. We saw no difference in outcomes between VDZ and UST for any subpopulation.

### 3.4. Biologic therapies in different treatment lines

To further inform drug sequencing decisions, we explored the comparative effectiveness of different biologic classes when used at different positions in treatment sequences.

#### 3.4.1. Ulcerative colitis

In total, 1302 evaluable patients received VDZ as first- [*n* = 626], second- [*n* = 540] or third-line therapy [*n* = 136] [Supplementary Table 9, Supplementary Figure 9]. VDZ showed similar effectiveness in each line of therapy up to 3 years of follow-up [Supplementary Figure 10].

We also assessed the effectiveness of anti-TNF agents when used as first- compared to second-line therapy after failure of VDZ. In total, 3390 evaluable patients with UC received either first-line anti-TNF therapy [*n* = 3346] or second-line anti-TNF therapy after failure of VDZ [*n* = 44] [Supplementary Table 10 and Supplementary Figure 11]. There was no significant difference in effectiveness when anti-TNF was used as first-line or second-line therapy after VDZ over 1 year of follow-up [Supplementary Figure 12].

#### 3.4.2. Crohn’s disease

**Figure 6.**
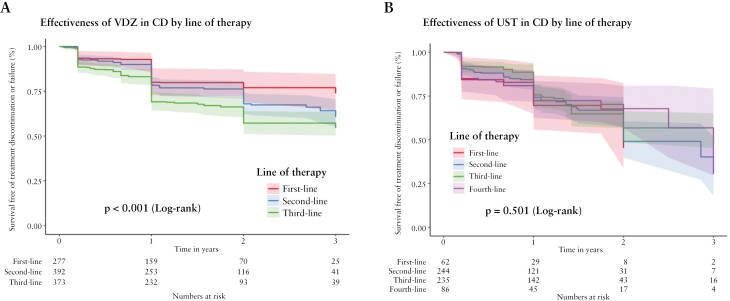
Effectiveness of therapy in Crohn’s disease [CD] for individual biologics according to line of therapy. Kaplan–Meier plots depict survival free of treatment discontinuation or failure after inverse probability of treatment weighting adjustment for patients with CD treated for up to 3 years, according to whether the biologic was used prior to any other biologic [first line, red], after one previous biologic [second line, blue], after two other prior biologics [third line, green], or after three prior biologic therapies [fourth line, purple]. [A] Use of vedolizumab in different lines of therapy. [B] Use of ustekinumab in different lines of therapy. Log-rank *p* values as shown.

Baseline characteristics for patients with CD who received VDZ as first-, second-, third- and fourth-line therapy are detailed in Supplementary Table 11. Physicians tended to prescribe VDZ as first-line therapy to older patients with uncomplicated disease without perianal or upper gastrointestinal tract involvement, as compared to use of anti-TNFs or use of VDZ in later lines of therapy. The effectiveness of VDZ treatment was superior when used as the first-line compared to later lines of therapy [Supplementary Figure 13; Figure 6A, *p* < 0.001].

We performed a similar analysis for UST. Again, patients treated early with UST tended to be older with less perianal involvement, although IPTW largely corrected for imbalances [Supplementary Table 12 and Supplementary Figure 14]. IPTW Kaplan–Meier analysis suggested similar effectiveness across different lines of therapy [Figure 6B, *p* = 0.501].

Only 17 patients received anti-TNF for CD as second-line therapy after prior failure of a non-anti-TNF biologic, reflecting UK reimbursement practice, and hence we did not assess anti-TNF usage by line of therapy in CD.

Finally, we assessed the impact of concomitant use of an immunomodulator across all biologics in both UC and CD. For IFX in both UC and CD and for ADA in CD, concomitant use of an immunomodulator was associated with a significant increase in drug effectiveness. For ADA in UC, as well as VDZ in both UC and CD and for UST in CD, there was no association of concomitant immunomodulator use with improved drug effectiveness [Supplementary Figure 15].

## 4. Discussion

We have presented data on the real-world use and long-term outcomes of biologic therapy in 13 222 patients and have provided evidence that challenges key current orthodoxies in treatment selection.

Despite the licensing of new biologic drugs for IBD and increasing access to biologics across many healthcare systems,^12–16^ anti-TNFs have largely retained their position as preferred first-line advanced therapies for IBD.^17^ Major drivers have been the licensing of cheaper biosimilar anti-TNF agents and extensive experience with these drugs. The risk is that physicians lock into this prescribing practice even as the evidence for alternative strategies emerges and indeed the costs of newer biologics themselves come down, as competition increases and biosimilars are introduced.

Selection of a first-line biologic therapy would ideally be informed by high-quality evidence drawn from head-to-head short- and long-term comparisons of efficacy, safety, health-economic, and quality of life outcomes. In reality, long-term outcomes are hard to measure in deliverable clinical trials. Furthermore, patients recruited to clinical trials and clinical practice within those trials may not best represent ‘real-world’ situations. This leaves an important data gap and underlines the need for robust, large-scale cohort studies.

In the present study, we obtained data on the effectiveness of biologics prescribed in over 13 000 patients with UC and CD, managed across multiple centres and with long-term follow-up. We used a composite measure of continued drug effectiveness based upon continuous receipt of a drug, with ongoing physician-reported effectiveness and without treatment discontinuation or any event indicative of treatment failure, including resectional/defunctioning bowel surgery. Additionally, we used a well-accepted statistical technique [IPTW] coupled with detailed phenotype collection to adjust for baseline variation in patient populations.

In UC, we found that VDZ was superior to anti-TNF over 5 years of follow-up. IFX was statistically superior to other anti-TNF agents but inferior to VDZ. The randomized trial literature for licensed drugs tested in head-to-head studies in UC consists of a single trial [VARSITY], which demonstrated the superiority of VDZ to ADA for the treatment of UC at standard doses over 1 year.^5^ Importantly, this benefit appeared confined to patients receiving first-line biologic therapy. In our study, VDZ was superior to ADA in both first- and second-line usage [after IFX failure]. We included 519 patients with previous IFX failure and up to 3 years of follow-up, dose-escalation of both drugs being allowed. This compares to 160 such patients followed up to 1 year in VARSITY wherein dose-escalation was prohibited. VARSITY also did not address the question of relative efficacy between VDZ and IFX. We were thus able to identify a signal for superiority of VDZ over IFX in both first-line usage and after ADA failure.

Our findings align with previous, smaller cohort studies that examined biologic selection in UC.^18–20^In a single centre retrospective study, Moens et al. showed superiority, in terms of treatment persistence as well as endoscopic remission, of VDZ over ADA as first-line agents in 109 biologic-naïve patients with moderate to severe UC.^18^ Similarly, an Australian prospective population-based study included 420 patients with moderate to severe UC who received either first-line IFX [*n* = 251] or VDZ [*n* = 169] with 774 patient-years of follow-up.^19^ VDZ used as a first-line agent had a significantly higher rate of persistence compared to IFX. Comparisons in second-line usage did not show a significant difference, but were based on a total of just 75 patients. A retrospective multicentre study using propensity score matching between groups showed that treatment persistence was significantly higher at 24 months with VDZ [*n* = 300] compared to anti-TNF agents [*n* = 296].^20^ However, it is notable that network meta-analyses of randomized trials^21,22^ did not demonstrate different efficacy between first-line VDZ and IFX in UC, perhaps reflecting the limitations of network meta-analysis or sparse trial data. Additionally, due to study designs, these network meta-analyses must compare efficacy during induction and maintenance therapy separately, whereas our analysis compares ‘treat-through’ outcomes over 5 years of follow-up.

In CD our data suggested similar effectiveness of IFX, ADA, and VDZ in luminal CD, but superiority of IFX over ADA in perianal disease. This supports international guidelines and aligns with both randomized clinical trials and other retrospective studies,^12,20,23,24^ but our findings extend follow-up to 10 years—which is important given the need to consider long-term outcomes in prescribing decisions.

Prescribing after anti-TNF failure is a common scenario in both UC and CD. In our study, around 25% of patients experienced treatment failure within 1 year of starting a first-line biologic for either UC or CD. Our outcome data inform second-line and sequential biologic prescribing.

Guidelines suggest that the mode of anti-TNF failure informs subsequent treatment decisions: for PNR, switching to a biologic with a different mechanism of action, while for NPNR, switching to a different anti-TNF.^1,25^ However, these strategies are based upon scientific inference, not clinical trial or real-world data. Importantly, they do not adjust for differences in efficacy between biologics in respective diseases. In our cohort, for patients with UC experiencing NPNR with IFX or ADA, switching to VDZ was more effective than an alternative anti-TNF. Likewise in CD, after NPNR to anti-TNF, switching to non-anti-TNF therapy was more effective than an alternative anti-TNF. These findings challenge ‘text-book’ pharmacokinetic management algorithms and reinforce the need for further data in this area. In the case of PNR with anti-TNF therapy, the differences between a second anti-TNF and a non-anti-TNF biologic were less clear in both UC and CD, although these findings should be interpreted in the context of smaller sample sizes in the PNR group. Nevertheless, after PNR to IFX in UC, ADA was clearly inferior to VDZ, whilst after PNR to ADA in UC, IFX and VDZ appeared equally effective. These findings probably reflect the relatively limited effectiveness of ADA in UC.

In patients with CD unresponsive to anti-TNF treatment, we observed similar effectiveness of UST and VDZ. Most earlier cohort studies have suggested superior effectiveness for UST over VDZ^7,26–28^ but had shorter follow-up and much smaller cohorts than the present study. Indeed, our findings align with two more recent, similarly sized cohort studies.^29,30^ Furthermore, indirect comparison of clinical trials using network meta-analysis has not demonstrated significant differences between VDZ and UST after failure of anti-TNF therapy.^31^ We saw no significant difference in effectiveness between VDZ and UST according to disease location, in line with registration trials.^32,33^ Taken together, these findings suggest that UST and VDZ offer similar effectiveness when used as second-line therapy in CD after anti-TNF failure, that both may offer superior effectiveness to a second anti-TNF in instances of NPNR, and that disease distribution should not determine treatment decisions.

Additional important questions relate to whether a drug’s effectiveness varies according to its position in the treatment sequence. In keeping with registrational trial data^34^ we found that VDZ was similarly effective in UC regardless of its position. Also in UC, anti-TNF therapies appeared to perform similarly when used as first-line or after failure of VDZ, a finding impossible to test in registrational trials. Given our evidence that VDZ outperformed both IFX and ADA in first- and second-line use in UC there is a case for positioning VDZ earlier in UC management, subject to appropriate health economic assessment. Currently, long-term health economic assessments of the impact of different biologic selections are limited and in many healthcare systems, payers may dictate payment according to selection of drugs with lower acquisition costs. Where economic considerations position anti-TNF therapies first in UC, our data and those of others^12,35,36^ support second-line usage of VDZ, regardless of mode of failure of anti-TNF.

In contrast, but also in accordance with trial data,^33,37^ VDZ appeared less effective in CD when used later in treatment sequences. This was not the case for UST, which contrasts with trial data.^32^ This may reflect superior assessment of performance of UST in patients with prior anti-TNF therapy outside the stringent conditions of clinical trials, in line with other cohort studies.^38–40^

The main strength of our study is that it is based on the large, nationwide cohort within the well-validated and curated UK IBD BioResource, providing real-world evidence in biologic sequencing with long durations of follow-up across a range of commonly encountered clinical scenarios including those not well represented in clinical trials. We used a more comprehensive definition of treatment effectiveness than prior studies,^12,19^ incorporating clinically assessed effectiveness and surgery alongside persistence on the drug. Our large cohort size also allowed us to consider several clinical scenarios and patient subpopulations not previously well addressed.

Study limitations include the risk of recall bias inherent in retrospective studies. In particular, although we were able to adjust for intravenous corticosteroid exposure, data on oral corticosteroid usage proved to be unreliable on data validation and we did not include these in our baseline covariates or in our assessment of treatment failure. Although on-licence dose escalation and therapeutic drug monitoring was available in most participating sites, we lacked data on drug and anti-drug antibody levels. Our assessment based on mode of failure could therefore not directly account for whether pharmacokinetic and immunogenicity-guided drug selection might offer advantages. Whilst biologic sequences used in this study reflect real-world practice, our results are necessarily limited by conventional prescribing patterns; thus, for example, we lack data on use of second-line anti-TNF therapies in CD after non-anti-TNF biologics. At times IPTW failed to adjust for differences in baseline covariates, but we did identify these and performed sensitivity analyses to confirm significant results. Additional covariates might have affected treatment selection and/or outcome that we have not identified/adjusted for. The assessment of failure was a clinical one, and whilst endoscopic outcomes may have informed decision-making, the clinician assessment of treatment failure lacks formal, documented endoscopic outcomes. Furthermore, physicians might set different thresholds for drug cessation according to how many treatments have failed previously, potentially complicating our analyses comparing effectiveness across different lines of therapy. Finally, due to timelines of data acquisition, we lacked sufficient patients treated with first-line UST for either CD or UC as well as for other new biologics and small molecule therapies.

## 5. Conclusion

Based on analysis of real-world data from the nationwide UK IBD BioResource, we assessed the effectiveness of biological therapies across a range of clinical scenarios commonly encountered when treating patients with IBD. Taken together, these results provide valuable insights into biologic drug selection and sequencing in the treatment of IBD.

## Supplementary Material

jjad203_suppl_Supplementary_Tables

jjad203_suppl_Supplementary_Figures

## Data Availability

The data underlying this article will be shared at aggregate/population level on reasonable request to the corresponding author. Patient-level data underlying this study are available to researchers subject to the access processes of the UK IBD BioResource, detailed further at https://www.ibdbioresource.nihr.ac.uk/index.php/resources/

## Appendix

UK IBD BioResource Investigators

Christopher Alexakis, Mohammed Allah-Ditta, Richard Appleby, Bijay Baburajan, Michelle Baker-Moffatt, Tyara Banerjee, Paul Banim, John Beckly, Roisin Bevan, Stuart Bloom, Monica Bose, Elaine Brinkworth, Johanne Brooks, Deborah Butcher, Jeffrey Butterworth, Monica Chan, Katie Clark, Andrew Cole, Joseph Collum, Rachel Cooney, Fraser Cummings, Albert Davies, Aminda De Silva, John DeCaestecker, Anjan Dhar, Stacey Duffy, Dharmaraj Durai, Cathryn Edwards, Stephen Foley, Tessa Glazebrook, John Gordon, Michael Grimes, Anton Gunasekera, Laura Hancock, Mina Hanna, Ailsa Hart, Gini Hay, David Hobday, Patricia Hooper, Mark Jarvis, Babur Javaid, Matthew Johnson, Lijo Joy, Rzwan Kassam, Nick Kennedy, Alexandra Kent, Klaartje Bel Kok, Konrad Koss, Nicola Lancaster, Jonathan Landy, Charlie Lees, Wendy Lewis, Stephen Lewis, Andy Li, Alan Lobo, Juliette Loehry, Chris Macdonald, Christopher Macdonald, George Macfaul, Zahid Mahmood, Dina Mansour, Simon McLaughlin, John McLaughlin, Yin Miao, Ajay Muddu, Charles Murray, Chuka Nwokolo, Susan O’Sullivan, Abby Oglesby, Simon Panter, Vinod Patel, Linda Patterson, Ruth Penn, Anne Phillips, Kath Phillis, Richard Pollok, Sam Powles, Cathryn Preston, Monira Rahman, Arvind Ramadas, John Ramage, Subramaniam Ramakrishnan, Jack Satsangi, John Saunders, Glyn Scott, Shaji Sebastian, Christian Selinger, Sherif Shabana, Rakesh Shah, Dan Sharpstone, Sophy Shedwell, Christopher Sheen, Richard Shenderey, Achuth Shenoy, Alison Simmons, Salil Singh, Leena Sinha, Ganesh Sivaji, Melissa Smith, Paul Smith, Katherine Smith, Helen Steed, Alan Steel, Byron Theron, Jude Tidbury, Theresa Tindall, Mark Tremelling, Deven Vani, Ajay Verma, Gareth Walker, Ben Warner, Alastair Watson, Emma Wesley, Alan Wiles, Joy Wilkins, Horace Williams.

### Supplementary Data

Supplementary data are available online at *ECCO-JCC* online.
