# Supplementary material for: Biologic Therapy for Inflammatory Bowel Disease: Real-World Comparative Effectiveness and Impact of Drug Sequencing in 13 222 Patients within the UK IBD BioResource
Source: J Crohns Colitis. 2023 Dec 2;18(6):790–800. doi: 10.1093/ecco-jcc/jjad203 (PMC11147798; doi:10.1093/ecco-jcc/jjad203)
Supplement: jjad203_suppl_Supplementary_Tables [file jjad203_suppl_supplementary_tables.pdf]

Supplementary tables

| Baseline Characteristics | **Infliximab**, (n = 2339)[1] | **Adalimumab**, (n = 864)[1] | **Golimumab**, (n = 143)[1] | **Vedolizumab**, (n =621)[1] | **p-value**[2] |
|---|---|---|---|---|---|
| **Sex, female N (%)** | 1,102 (47%) | 416 (48%) | 60 (42%) | 254 (41%) | **0.017** |
| **Age at baseline (years), median (IQR)** | 37 (27, 50) | 40 (29, 53) | 41 (30, 55) | 48 (33, 64) | **<0.001** |
| **Disease extent, N (%)** | | | | | 0.40 |
| Extended | 845 (40%) | 335 (43%) | 48 (38%) | 234 (42%) | |
| Left sided | 1,274 (60%) | 451 (57%) | 80 (63%) | 319 (58%) | |
| Unknown | 220 | 78 | 15 | 68 | |
| **Disease duration (years), median (IQR)** | 9 (6, 15) | 10 (6, 16) | 10 (8, 17) | 11 (6, 17) | **<0.001** |
| Unknown | 3 | 3 | 1 | 2 | |
| **Combination treatment at baseline, N (%)** | 1,122 (48%) | 314 (36%) | 60 (42%) | 162 (26%) | **<0.001** |
| **Steroid use at baseline, N (%)** | 398 (17%) | 41 (4.7%) | 3 (2.1%) | 20 (3.2%) | **<0.001** |
| **History of EIM, N (%)**[3] | 603 (26%) | 223 (26%) | 51 (36%) | 212 (34%) | **<0.001** |
| **Smoking at diagnosis, N (%)** | 326 (15%) | 104 (13%) | 12 (9.1%) | 72 (13%) | 0.11 |
| Unknown | 168 | 72 | 11 | 45 | |

[1] n (%); Median (IQR)

[2] Pearson's Chi-squared test; Kruskal-Wallis rank sum test

[3] EIM, extraintestinal manifestations

Supplemental Table 1 – Baseline characteristics in patients with ulcerative colitis starting first-line biologic therapy

| **Baseline Characteristics** | **Infliximab**, (n = 5406)[1] | **Adalimumab**, (n = 3097)[1] | **Vedolizumab**, (n = 277)[1] | **p-value**[2] |
|---|---|---|---|---|
| **Age at baseline (years), median (IQR)** | 32 (23, 45) | 35 (25, 49) | 57 (38, 69) | **<0.001** |
| **Sex, female N (%)** | 2,773 (51%) | 1,648 (53%) | 144 (52%) | 0.23 |
| **Smoking at diagnosis, N (%)** | 1,662 (33%) | 1,019 (35%) | 79 (31%) | 0.068 |
| Unknown | 332 | 192 | 20 | |
| **Disease duration (years), median (IQR)** | 14 (8, 22) | 12 (8, 21) | 12 (6, 24) | **0.002** |
| Unknown | 22 | 6 | 1 | |
| **Disease extent, N (%)** | | | | **<0.001** |
| Ileal | 1,497 (29%) | 1,199 (40%) | 105 (39%) | |
| Colonic | 1,602 (31%) | 675 (23%) | 82 (31%) | |
| Ileo-colonic | 2,096 (40%) | 1,114 (37%) | 80 (30%) | |
| Unknown | 211 | 109 | 10 | |
| **Upper GI involvement, N (%)** | 473 (8.7%) | 260 (8.4%) | 18 (6.5%) | 0.39 |
| **Disease behavior, N (%)** | | | | **<0.001** |
| Inflammatory | 3,049 (60%) | 1,680 (57%) | 162 (67%) | |
| Stricturing | 1,255 (25%) | 886 (30%) | 63 (26%) | |
| Penetrating | 786 (15%) | 372 (13%) | 18 (7.4%) | |
| Unknown | 316 | 159 | 34 | |
| **Peri-anal disease, N (%)** | 2,308 (45%) | 917 (31%) | 71 (28%) | **<0.001** |
| Unknown | 280 | 172 | 21 | |
| **Prior intestinal resection, N (%)** | 1,648 (30%) | 1,098 (35%) | 88 (32%) | **<0.001** |
| **History of EIM, N (%)**[3] | 1,942 (36%) | 1,054 (34%) | 107 (39%) | 0.11 |
| **Prior peri-anal interventions, N (%)** | 643 (12%) | 193 (6.2%) | 12 (4.3%) | **<0.001** |
| **Combination treatment at baseline, N (%)** | 2,817 (52%) | 1,253 (40%) | 59 (21%) | **<0.001** |
| **Steroids use at baseline, N (%)** | 292 (5.4%) | 83 (2.7%) | 5 (1.8%) | **<0.001** |

[1] Median (IQR); n (%)

[2] Kruskal-Wallis rank sum test; Pearson's Chi-squared test

[3] EIM, extraintestinal manifestations

Supplemental Table 2 - Baseline characteristics in patients with Crohn's disease starting first-line biologic therapy

| **Baseline Characteristics** | **Infliximab**, (n = 3083)[1] | **Adalimumab**, (n = 2171)[1] | **Vedolizumab**, (n = 206)[1] | **p-value**[2] |
|---|---|---|---|---|
| **Age at baseline (years), median (IQR)** | 33 (23, 47) | 36 (26, 50) | 60 (43, 70) | **<0.001** |
| **Sex, female N (%)** | 1,635 (53%) | 1,184 (55%) | 105 (51%) | 0.42 |
| **Smoking at diagnosis, N (%)** | 966 (34%) | 727 (36%) | 57 (30%) | 0.11 |
| Unknown | 221 | 148 | 15 | |
| **Disease duration (years), median (IQR)** | 13 (8, 21) | 12 (7, 20) | 12 (6, 22) | **0.002** |
| Unknown | 15 | 5 | 1 | |
| **Disease extent, N (%)** | | | | **<0.001** |
| Ileal | 1,099 (37%) | 952 (46%) | 85 (43%) | |
| Colonic | 746 (25%) | 426 (20%) | 57 (29%) | |
| Ileo-colonic | 1,108 (38%) | 707 (34%) | 58 (29%) | |
| Unknown | 130 | 86 | 6 | |
| **Upper GI involvement, N (%)** | 272 (8.8%) | 174 (8.0%) | 14 (6.8%) | 0.40 |
| **Disease behavior, N (%)** | | | | **<0.001** |
| Inflammatory | 1,802 (62%) | 1,220 (59%) | 131 (70%) | |
| Stricturing | 771 (27%) | 634 (31%) | 46 (25%) | |
| Penetrating | 314 (11%) | 199 (9.7%) | 9 (4.8%) | |
| Unknown | 196 | 118 | 20 | |
| **Prior intestinal resection, N (%)** | 938 (30%) | 732 (34%) | 59 (29%) | **0.026** |
| **History of EIM, N (%)**[3] | 1,047 (34%) | 725 (33%) | 76 (37%) | 0.59 |
| **Combination treatment at baseline, N (%)** | 1,571 (51%) | 854 (39%) | 40 (19%) | **<0.001** |
| **Steroids use at baseline, N (%)** | 201 (6.5%) | 60 (2.8%) | 3 (1.5%) | **<0.001** |

[1] Median (IQR); n (%)

[2] Kruskal-Wallis rank sum test; Pearson's Chi-squared test

[3] EIM, extraintestinal manifestations

Supplemental Table 3 - Baseline characteristics in patients with Crohn's disease and no perianal involvement starting first-line biologic therapy

| **Baseline Characteristics** | **Infliximab**, (n = 2323)[1] | **Adalimumab**, (n = 926)[1] | **Vedolizumab**, (n = 71)[1] | **p-value**[2] |
|---|---|---|---|---|
| **Age at baseline (years), median (IQR)** | 31 (22, 43) | 32 (24, 44) | 48 (29, 64) | **<0.001** |
| **Sex, female N (%)** | 1,138 (49%) | 464 (50%) | 39 (55%) | 0.55 |
| **Smoking at diagnosis, N (%)** | 696 (31%) | 292 (33%) | 22 (33%) | 0.66 |
| Unknown | 111 | 44 | 5 | |
| **Disease duration (years), median (IQR)** | 15 (9, 22) | 15 (9, 22) | 16 (8, 28) | 0.45 |
| Unknown | 7 | 1 | 0 | |
| **Disease extent, N (%)** | | | | **<0.001** |
| Ileal | 398 (18%) | 247 (27%) | 20 (30%) | |
| Colonic | 856 (38%) | 249 (28%) | 25 (37%) | |
| Ileo-colonic | 988 (44%) | 407 (45%) | 22 (33%) | |
| Unknown | 81 | 23 | 4 | |
| **Upper GI involvement, N (%)** | 201 (8.7%) | 86 (9.3%) | 4 (5.6%) | 0.54 |
| **Disease behavior, N (%)** | | | | **0.003** |
| Inflammatory | 1,247 (57%) | 460 (52%) | 31 (54%) | |
| Stricturing | 484 (22%) | 252 (28%) | 17 (30%) | |
| Penetrating | 472 (21%) | 173 (20%) | 9 (16%) | |
| Unknown | 120 | 41 | 14 | |
| **Prior intestinal resection, N (%)** | 710 (31%) | 366 (40%) | 29 (41%) | **<0.001** |
| **History of EIM, N (%)**[3] | 895 (39%) | 329 (36%) | 31 (44%) | 0.17 |
| **Combination treatment at baseline, N (%)** | 1,246 (54%) | 399 (43%) | 19 (27%) | **<0.001** |
| **Steroid use at baseline, N (%)** | 91 (3.9%) | 23 (2.5%) | 2 (2.8%) | 0.12 |

[1] Median (IQR); n (%)

[2] Kruskal-Wallis rank sum test; Pearson's Chi-squared test; Fisher's exact test

[3] EIM, extraintestinal manifestations

Supplemental table 4 – Baseline characteristics in patients with Crohn's disease and perianal involvement starting first-line biologic therapy

| Baseline Characteristics | **Infliximab**, (n = 92)[1] | **Vedolizumab**, (n = 209)[1] | **p-value**[2] |
|---|---|---|---|
| **Sex, female N (%)** | 41 (45%) | 102 (49%) | 0.50 |
| **Age at baseline (years), median (IQR)** | 38 (30, 49) | 40 (30, 54) | 0.24 |
| **Disease extent, N (%)** | | | 0.58 |
| Extended | 36 (44%) | 77 (40%) | |
| Left sided | 46 (56%) | 114 (60%) | |
| Unknown | 10 | 18 | |
| **Disease duration (years), median (IQR)** | 10 (7, 16) | 10 (6, 16) | 0.73 |
| Unknown | 1 | 0 | |
| **Smoking at diagnosis, N (%)** | 8 (11%) | 28 (14%) | 0.42 |
| Unknown | 17 | 15 | |
| **Combination treatment at baseline, N (%)** | 36 (39%) | 72 (34%) | 0.44 |
| **Steroid use at baseline, N (%)** | 8 (8.7%) | 13 (6.2%) | 0.44 |
| **History of EIM, N (%)**[3] | 28 (30%) | 52 (25%) | 0.31 |
| **Reason for anti-TNF discontinuation prior to current treatment, N (%)** | | | 0.058 |
| Non primary non-response | 58 (63%) | 106 (51%) | |
| Primary non-response | 34 (37%) | 101 (49%) | |
| Unknown | 0 | 2 | |

[1] n (%); Median (IQR)

[2] Pearson's Chi-squared test; Wilcoxon rank sum test

[3] EIM, extraintestinal manifestations

Supplemental table 5 – Baseline characteristics in patients with ulcerative colitis who received second-line therapy with infliximab vs vedolizumab after adalimumab failure

| Baseline Characteristics | **Adalimumab**, (n = 229)[1] | **Vedolizumab**, (n = 290)[1] | **p-value**[2] |
|---|---|---|---|
| **Sex, female N (%)** | 131 (57%) | 137 (47%) | **0.024** |
| **Age at baseline (years), median (IQR)** | 38 (28, 51) | 37 (29, 52) | 0.90 |
| **Disease extent, N (%)** | | | 0.48 |
| Extended | 87 (41%) | 115 (44%) | |
| Left sided | 127 (59%) | 147 (56%) | |
| Unknown | 15 | 28 | |
| **Disease duration (years), median (IQR)** | 10 (7, 16) | 10 (6, 15) | 0.072 |
| **Smoking at diagnosis** | 39 (18%) | 36 (13%) | 0.13 |
| Unknown | 15 | 18 | |
| **Combination treatment at baseline, N (%)** | 83 (36%) | 108 (37%) | 0.82 |
| **Steroid use at baseline, N (%)** | 18 (7.9%) | 26 (9.0%) | 0.65 |
| **History of EIM, N (%)**[3] | 55 (24%) | 88 (30%) | 0.11 |
| **Reason for anti-TNF discontinuation prior to current treatment, N (%)** | | | **<0.001** |
| Non primary non-response | 173 (80%) | 178 (62%) | |
| Primary non-response | 42 (20%) | 110 (38%) | |
| Unknown | 14 | 2 | |

[1] n (%); Median (IQR)

[2] Pearson's Chi-squared test; Wilcoxon rank sum test

[3] EIM, extraintestinal manifestations

Supplemental table 6 – Baseline characteristics in patients with ulcerative colitis who received second-line therapy with adalimumab vs vedolizumab after infliximab failure

| Baseline Characteristics | Anti-TNF (Infliximab and Adalimumab), (n = 1925)[1] | Non-anti-TNF (Vedolizumab and Ustekinumab), (n = 614)[1] | p-value[2] |
|---|---|---|---|
| **Age at baseline (years), median (IQR)** | 33 (25, 44) | 38 (28, 52) | **<0.001** |
| **Sex, female N (%)** | 1,061 (55%) | 367 (60%) | **0.043** |
| **Smoking at diagnosis, N (%)** | 643 (36%) | 200 (35%) | 0.80 |
| Unknown | 116 | 42 | |
| **Disease duration (years), median (IQR)** | 16 (11, 22) | 13 (8, 21) | **<0.001** |
| Unknown | 8 | 0 | |
| **Disease extent, N (%)** | | | **0.003** |
| Ileal | 499 (27%) | 197 (33%) | |
| Colonic | 492 (26%) | 156 (26%) | |
| Ileo-colonic | 872 (47%) | 236 (40%) | |
| Unknown | 62 | 25 | |
| **Upper GI involvement, N (%)** | 223 (12%) | 47 (7.7%) | **0.006** |
| **Disease behavior, N (%)** | | | **0.011** |
| Inflammatory | 985 (54%) | 320 (57%) | |
| Stricturing | 535 (29%) | 177 (31%) | |
| Penetrating | 307 (17%) | 65 (12%) | |
| Unknown | 98 | 52 | |
| **Peri-anal disease, N (%)** | 886 (48%) | 213 (37%) | **<0.001** |
| Unknown | 98 | 37 | |
| **Prior intestinal resection, N (%)** | 797 (41%) | 213 (35%) | **0.003** |
| **History of EIM, N (%)**[3] | 804 (42%) | 253 (41%) | 0.81 |
| **Prior peri-anal interventions, N (%)** | 213 (11%) | 49 (8.0%) | **0.029** |
| **Combination treatment at baseline, N (%)** | 868 (45%) | 205 (33%) | **<0.001** |
| **Steroid use at baseline, N (%)** | 55 (2.9%) | 19 (3.1%) | 0.76 |
| **Reason for anti-TNF discontinuation prior to current treatment, N (%)** | | | 0.30 |
| Non primary non-response | 1,510 (80%) | 474 (78%) | |
| Primary non-response | 371 (20%) | 131 (22%) | |
| Unknown | 44 | 9 | |

[1] Median (IQR); n (%)

[2] Wilcoxon rank sum test; Pearson's Chi-squared test

[3] EIM, extraintestinal manifestations

Supplemental table 7 - Baseline characteristics in patients with Crohn's disease who received second-line therapy with non-anti-TNF vs an anti-TNF agent after first-line anti-TNF failure

| Baseline Characteristics | **Vedolizumab**, (n = 743)[1] | **Ustekinumab**, (n = 425)[1] | **p-value**[2] |
|---|---|---|---|
| **Age at baseline (years), median (IQR)** | 36 (28, 51) | 35 (27, 50) | 0.10 |
| **Sex, female N (%)** | 436 (59%) | 223 (52%) | **0.039** |
| **Smoking at diagnosis, N (%)** | 237 (34%) | 130 (33%) | 0.89 |
| Unknown | 39 | 34 | |
| **Disease duration (years), median (IQR)** | 15 (10, 22) | 14 (9, 22) | 0.21 |
| Unknown | 1 | 0 | |
| **Disease extent, N (%)** | | | **0.002** |
| Ileal | 188 (26%) | 132 (32%) | |
| Colonic | 214 (30%) | 85 (21%) | |
| Ileo-colonic | 311 (44%) | 193 (47%) | |
| Unknown | 30 | 15 | |
| **Upper GI involvement, N (%)** | 66 (8.9%) | 51 (12%) | 0.088 |
| **Disease behavior, N (%)** | | | **0.002** |
| Inflammatory | 389 (57%) | 185 (46%) | |
| Stricturing | 200 (29%) | 152 (38%) | |
| Penetrating | 93 (14%) | 63 (16%) | |
| Unknown | 61 | 25 | |
| **Peri-anal disease, N (%)** | 298 (42%) | 185 (46%) | 0.26 |
| Unknown | 41 | 22 | |
| **Prior intestinal resection, N (%)** | 300 (40%) | 182 (43%) | 0.41 |
| **History of EIM, N (%)**[3] | 312 (42%) | 184 (43%) | 0.66 |
| **Prior peri-anal interventions, N (%)** | 73 (9.8%) | 33 (7.8%) | 0.24 |
| **Combination treatment at baseline, N (%)** | 266 (36%) | 144 (34%) | 0.51 |
| **Steroid use at baseline, N (%)** | 26 (3.5%) | 16 (3.8%) | 0.81 |
| **Reason for anti-TNF discontinuation prior to current treatment, N (%)** | | | 0.11 |
| Non primary non-response | 530 (73%) | 322 (77%) | |
| Primary non-response | 197 (27%) | 95 (23%) | |
| Unknown | 16 | 8 | |
| **Therapy sequence, N (%)** | | | 0.64 |
| Second line | 388 (52%) | 228 (54%) | |
| Third line | 355 (48%) | 197 (46%) | |

[1] Median (IQR); n (%)

[2] Wilcoxon rank sum test; Pearson's Chi-squared test

[3] EIM, extraintestinal manifestations

Supplemental table 8 - Baseline characteristics in patients with Crohn's disease who received second- or third-line therapy with vedolizumab vs ustekinumab after one or two anti-TNF agent

failures

| Baseline Characteristics | **VDZ first line**, (n = 626)[1] | **VDZ second line**, (n = 540)[1] | **VDZ third line**, (n = 136)[1] | **p-value**[2] |
|---|---|---|---|---|
| **Sex, female N (%)** | 256 (41%) | 257 (48%) | 64 (47%) | 0.057 |
| **Disease duration (years), median (IQR)** | 11 (6, 18) | 10 (6, 16) | 10 (8, 15) | 0.25 |
| Unknown | 2 | 1 | 0 | |
| **Age at baseline (years), median (IQR)** | 47 (33, 64) | 38 (29, 53) | 42 (29, 53) | **<0.001** |
| **Combination treatment at baseline, N (%)** | 163 (26%) | 200 (37%) | 40 (29%) | **<0.001** |
| **History of EIM, N (%)**[3] | 214 (34%) | 152 (28%) | 44 (32%) | 0.084 |
| **Steroid use at baseline, N (%)** | 21 (3.4%) | 32 (5.9%) | 5 (3.7%) | 0.094 |
| **Disease extent, N (%)** | | | | 0.81 |
| Extended | 236 (42%) | 207 (42%) | 47 (39%) | |
| Left sided | 321 (58%) | 285 (58%) | 73 (61%) | |
| Unknown | 69 | 48 | 16 | |

[1] n (%); Median (IQR)

[2] Pearson's Chi-squared test; Kruskal-Wallis rank sum test

[3] EIM, extraintestinal manifestations

Supplemental Table 9: Baseline characteristics in patients with ulcerative colitis who received vedolizumab as first-, second- or third-line therapy

| **Baseline Characteristics** | **Anti-TNF first line**, (n = 3346)[1] | **Anti-TNF second line**, (n = 44)[1] | **p-value**[2] |
|---|---|---|---|
| **Sex, female N (%)** | 1,578 (47%) | 19 (43%) | 0.60 |
| **Disease duration (years), median (IQR)** | 9 (6, 15) | 10 (6, 13) | 0.96 |
| Unknown | 7 | 0 | |
| **Age at baseline (years), median (IQR)** | 38 (28, 51) | 40 (31, 56) | 0.18 |
| **Combination treatment at baseline, N (%)** | 1,496 (45%) | 14 (32%) | 0.087 |
| **History of EIM, N (%)**[3] | 877 (26%) | 20 (45%) | **0.004** |
| **Steroid use at baseline, N (%)** | 442 (13%) | 2 (4.5%) | 0.091 |
| **Disease extent, N (%)** | | | 0.063 |
| Extended | 1,228 (40%) | 22 (55%) | |
| Left sided | 1,805 (60%) | 18 (45%) | |
| Unknown | 313 | 4 | |

[1] n (%); Median (IQR)

[2] Pearson's Chi-squared test; Wilcoxon rank sum test

[3] EIM, extraintestinal manifestations

Supplemental table 10: Baseline characteristics in patients with ulcerative colitis who received first-line anti-TNF agents vs second-line anti-TNF agents after vedolizumab failure

| Baseline Characteristics | **VDZ first line**, (n = 277)[1] | **VDZ second line**, (n = 392)[1] | **VDZ third line**, (n = 373)[1] | **VDZ fourth line**, (n = 25)[1] | **p-value**[2] |
|---|---|---|---|---|---|
| **Age at baseline (years), median (IQR)** | 57 (38, 69) | 39 (29, 52) | 35 (27, 48) | 26 (23, 43) | **<0.001** |
| **Sex, female N (%)** | 144 (52%) | 247 (63%) | 199 (53%) | 7 (28%) | **<0.001** |
| **Smoking at diagnosis, N (%)** | 79 (31%) | 129 (35%) | 114 (32%) | 4 (17%) | 0.26 |
| Unknown | 20 | 22 | 20 | 1 | |
| **Disease duration (years), median (IQR)** | 12 (6, 24) | 13 (8, 22) | 17 (11, 22) | 13 (10, 19) | **<0.001** |
| Unknown | 1 | 0 | 1 | 0 | |
| **Disease extent, N (%)** | | | | | **<0.001** |
| Ileal | 105 (39%) | 112 (30%) | 86 (24%) | 5 (24%) | |
| Colonic | 82 (31%) | 113 (30%) | 102 (28%) | 3 (14%) | |
| Ileo-colonic | 80 (30%) | 149 (40%) | 172 (48%) | 13 (62%) | |
| Unknown | 10 | 18 | 13 | 4 | |
| **Upper GI involvement, N (%)** | 18 (6.5%) | 22 (5.6%) | 48 (13%) | 4 (16%) | **<0.001** |
| **Disease behavior, N (%)** | | | | | |
| Inflammatory | 162 (67%) | 215 (60%) | 184 (54%) | 11 (48%) | |
| Stricturing | 63 (26%) | 101 (28%) | 105 (31%) | 9 (39%) | |
| Penetrating | 18 (7.4%) | 41 (11%) | 53 (15%) | 3 (13%) | |
| Unknown | 34 | 35 | 31 | 2 | |
| **Peri-anal disease, N (%)** | 71 (28%) | 129 (35%) | 176 (50%) | 13 (59%) | **<0.001** |
| Unknown | 21 | 25 | 19 | 3 | |
| **Prior intestinal resection, N (%)** | 88 (32%) | 133 (34%) | 178 (48%) | 8 (32%) | **<0.001** |
| **History of EIM, N (%)**[3] | 107 (39%) | 153 (39%) | 166 (45%) | 10 (40%) | 0.37 |
| **Prior peri-anal interventions, N (%)** | 12 (4.3%) | 35 (8.9%) | 39 (10%) | 4 (16%) | **0.010** |
| **Combination treatment at baseline, N (%)** | 59 (21%) | 133 (34%) | 137 (37%) | 11 (44%) | **<0.001** |
| **steroid use at baseline, N (%)** | 5 (1.8%) | 11 (2.8%) | 15 (4.0%) | 2 (8.0%) | 0.15 |

[1] Median (IQR); n (%)

[2] Kruskal-Wallis rank sum test; Pearson's Chi-squared test; Fisher's exact test

[3] EIM, extraintestinal manifestations

Supplemental Table 11: Baseline characteristics in patients with Crohn's disease who received vedolizumab as first-, second-, third- or fourth-line therapy

| Baseline Characteristics | **UST first line**, (n = 62)[1] | **UST second line**, (n = 244)[1] | **UST third line**, (n = 235)[1] | **UST fourth line**, (n = 86)[1] | **p-value**[2] |
|---|---|---|---|---|---|
| **Age at baseline (years), median (IQR)** | 42 (29, 56) | 37 (28, 52) | 34 (26, 46) | 32 (25, 46) | **0.003** |
| **Sex, female N (%)** | 33 (53%) | 133 (55%) | 122 (52%) | 43 (50%) | 0.89 |
| **Smoking at diagnosis, N (%)** | 23 (43%) | 81 (36%) | 72 (33%) | 24 (29%) | 0.34 |
| Unknown | 8 | 21 | 15 | 3 | |
| **Disease duration (years), median (IQR)** | 12 (7, 20) | 13 (7, 22) | 16 (11, 23) | 18 (12, 22) | **<0.001** |
| **Disease extent, N (%)** | | | | | **<0.001** |
| Ileal | 26 (46%) | 98 (41%) | 51 (22%) | 17 (20%) | |
| Colonic | 11 (19%) | 47 (20%) | 54 (24%) | 16 (19%) | |
| Ileo-colonic | 20 (35%) | 92 (39%) | 122 (54%) | 52 (61%) | |
| Unknown | 5 | 7 | 8 | 1 | |
| **Upper GI involvement, N (%)** | 6 (9.7%) | 26 (11%) | 28 (12%) | 18 (21%) | 0.075 |
| **Disease behavior, N (%)** | | | | | **0.017** |
| Inflammatory | 31 (58%) | 119 (52%) | 94 (42%) | 31 (38%) | |
| Stricturing | 15 (28%) | 84 (37%) | 86 (38%) | 35 (43%) | |
| Penetrating | 7 (13%) | 24 (11%) | 46 (20%) | 16 (20%) | |
| Unknown | 9 | 17 | 9 | 4 | |
| **Peri-anal disease, N (%)** | 18 (31%) | 89 (39%) | 119 (53%) | 47 (59%) | **<0.001** |
| Unknown | 4 | 16 | 11 | 7 | |
| **Prior intestinal resection, N (%)** | 19 (31%) | 88 (36%) | 116 (49%) | 53 (62%) | **<0.001** |
| **History of EIM, N (%)**[3] | 20 (32%) | 108 (44%) | 103 (44%) | 42 (49%) | 0.24 |
| **Prior peri-anal interventions, N (%)** | 3 (4.8%) | 14 (5.7%) | 26 (11%) | 8 (9.3%) | 0.13 |
| **Combination treatment at baseline, N (%)** | 19 (31%) | 77 (32%) | 75 (32%) | 27 (31%) | >0.99 |
| **steroid use at baseline, N (%)** | 1 (1.6%) | 8 (3.3%) | 9 (3.8%) | 3 (3.5%) | 0.92 |

[1] Median (IQR); n (%)

[2] Kruskal-Wallis rank sum test; Pearson's Chi-squared test; Fisher's exact test

[3] EIM, extraintestinal manifestations

Supplemental table 12: Baseline characteristics in patients with Crohn's disease who received ustekinumab as first-, second-, third- or fourth-line therapy
