# Supplementary material for: Biologic Therapy for Inflammatory Bowel Disease: Real-World Comparative Effectiveness and Impact of Drug Sequencing in 13 222 Patients within the UK IBD BioResource
Source: J Crohns Colitis. 2023 Dec 2;18(6):790–800. doi: 10.1093/ecco-jcc/jjad203 (PMC11147798; doi:10.1093/ecco-jcc/jjad203)
Supplement: jjad203_suppl_Supplementary_Figures [file jjad203_suppl_supplementary_figures.pdf]

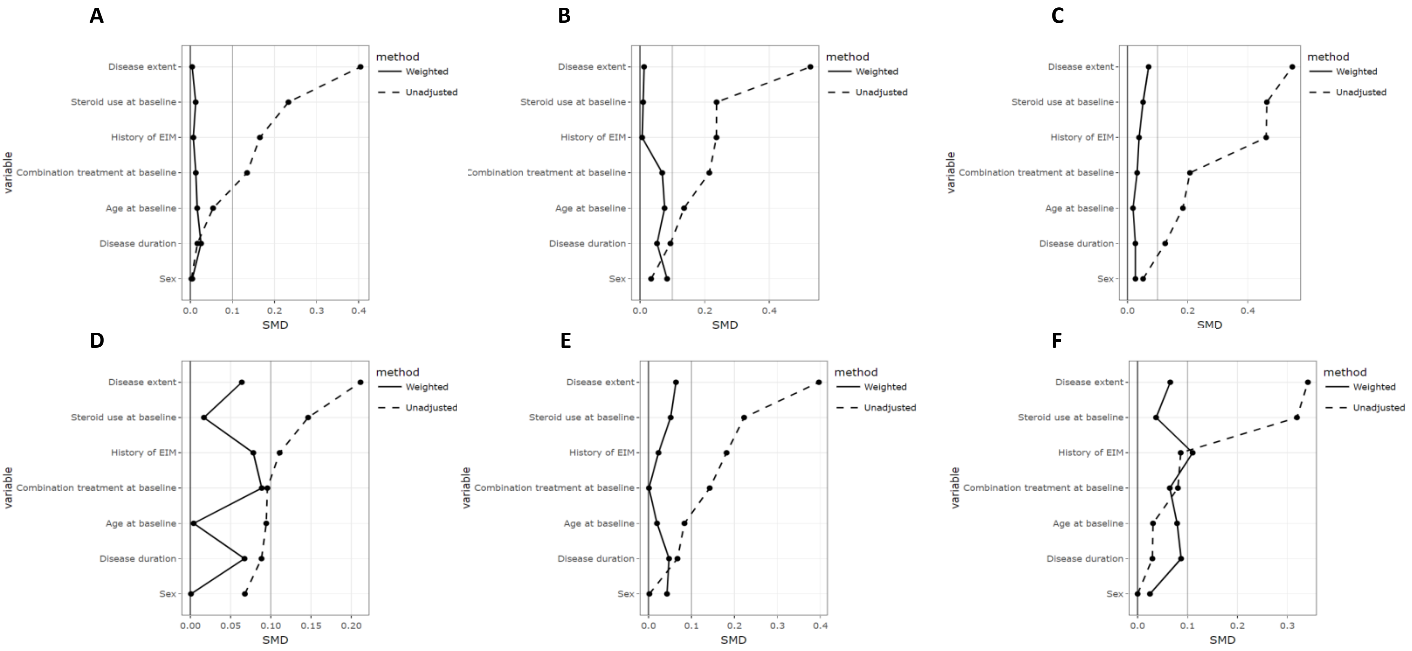

Supplemental Figure 1: SMD in baseline covariates between patients with UC who started a first-line biologic therapy. SMD of baseline confounding covariates that were included for estimating the propensity scores before (dotted lines) and after weighting (solid lines). Solid vertical line corresponds to SMD of 10% indicative of between group balance after weighting for comparisons between groups of patients starting relevant comparator treatments: (A) IFX vs ADA; (B) IFX vs GLM; (C) IFX vs VDZ; (D) ADA vs GLM; (E) ADA vs VDZ; (F) GLM vs VDZ.

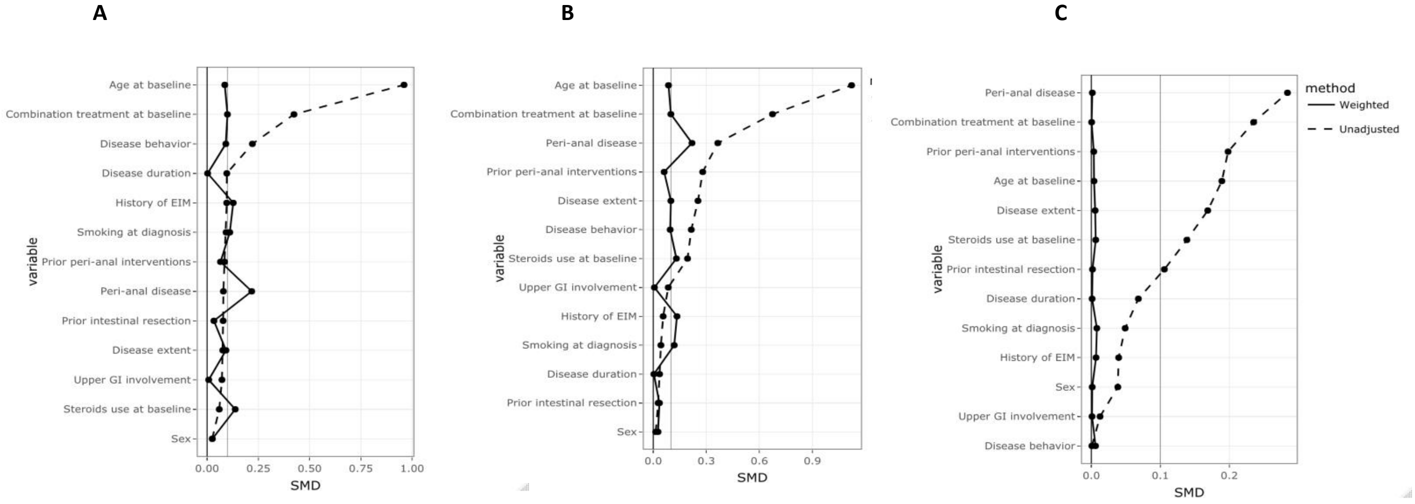

Supplemental Figure 2: SMD in baseline covariates between patients with CD who started a first-line biologic therapy. SMD of baseline confounding covariates that were included for estimating the propensity scores before (dotted lines) and after weighting (solid lines). Solid vertical line corresponds to SMD of 10% indicative of between group balance after weighting for comparisons between groups of patients starting relevant comparator treatments: (A) ADA vs VDZ; (B) IFX vs VDZ; (C) IFX vs ADA.

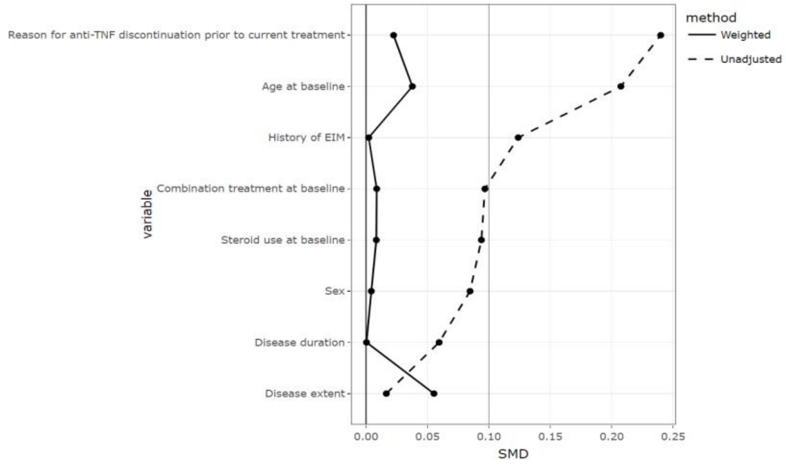

Supplemental Figure 3: SMD in baseline covariates between patients with UC who started IFX vs VDZ therapy after failure of ADA. SMD of baseline confounding covariates that were included for estimating the propensity scores before (dotted line) and after weighting (solid line). Solid vertical line corresponds to SMD of 10% indicative of between group balance after weighting for comparisons between groups of patients.

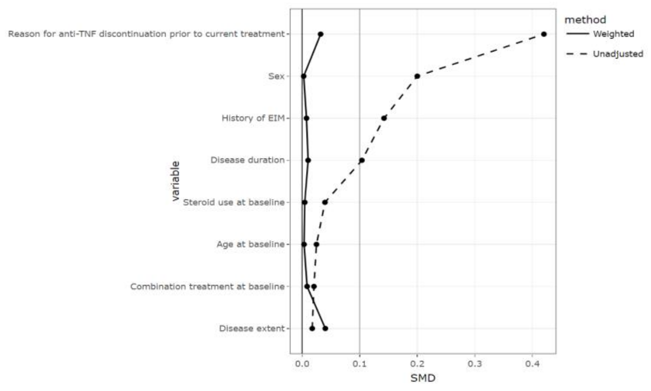

Supplemental Figure 4: SMD in baseline covariates between patients with UC who started ADA vs VDZ therapy after failure of IFX. SMD of baseline confounding covariates that were included for estimating the propensity scores before (dotted line) and after weighting (solid line). Solid vertical line corresponds to SMD of 10% indicative of between group balance after weighting for comparisons between groups of patients.

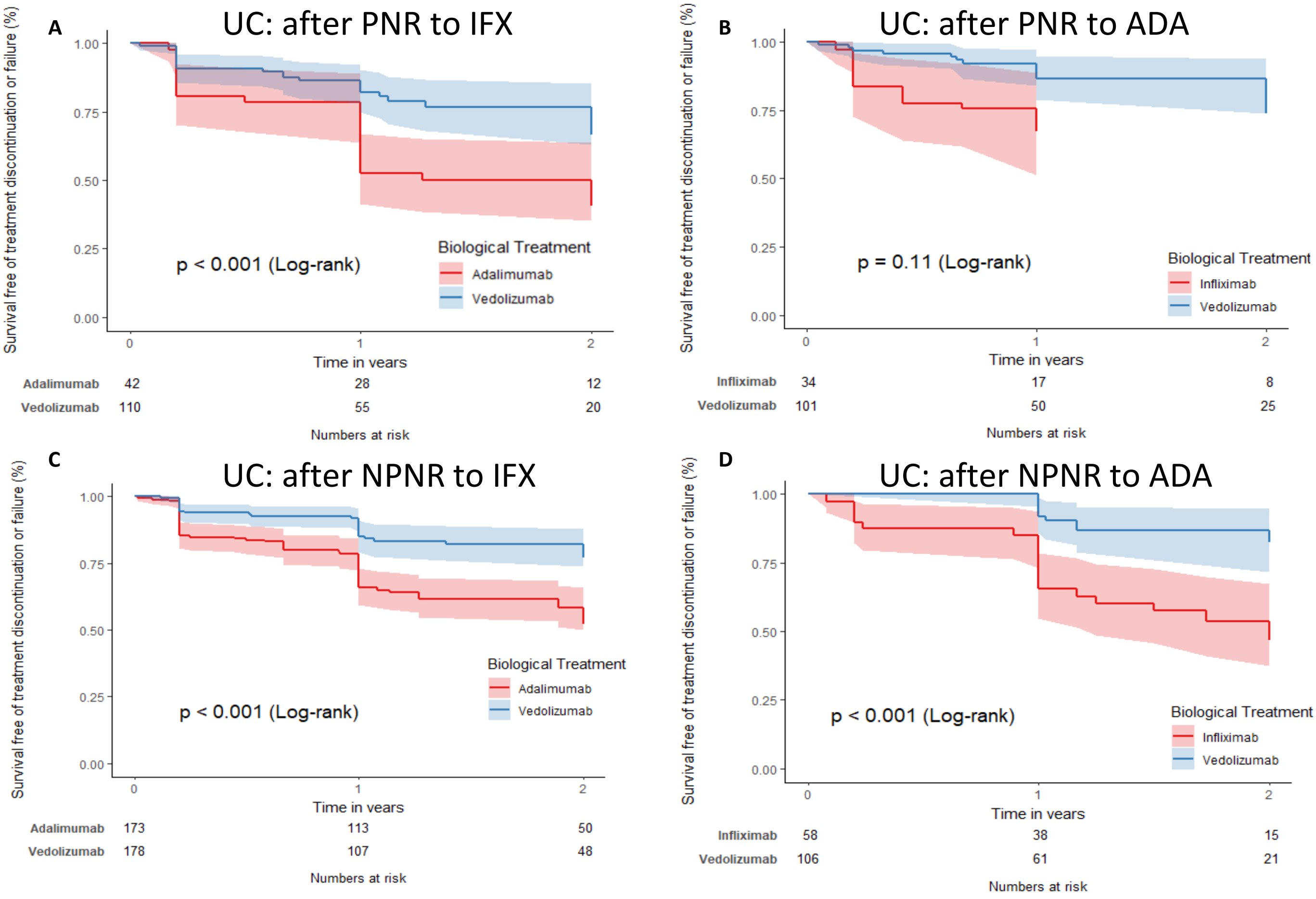

Supplemental Figures 5: Effectiveness of therapy in UC after failure of first-line anti-TNF according to mode of failure. Kaplan-Meier plots depict survival free of treatment discontinuation or failure after IPTW adjustment for patients with UC treated for up to 2 years. **(A)** Use of ADA (red) compared to VDZ (blue) as second line therapy after PNR to IFX. **(B)** Use of IFX (red) compared to VDZ (blue) as second line therapy after PNR to ADA. **(C)** Use of ADA (red) compared to VDZ (blue) as second line therapy after NPNR to IFX. **(D)** Use of IFX (red) compared to VDZ (blue) as second line therapy after NPNR to ADA. Log-rank p values as shown.

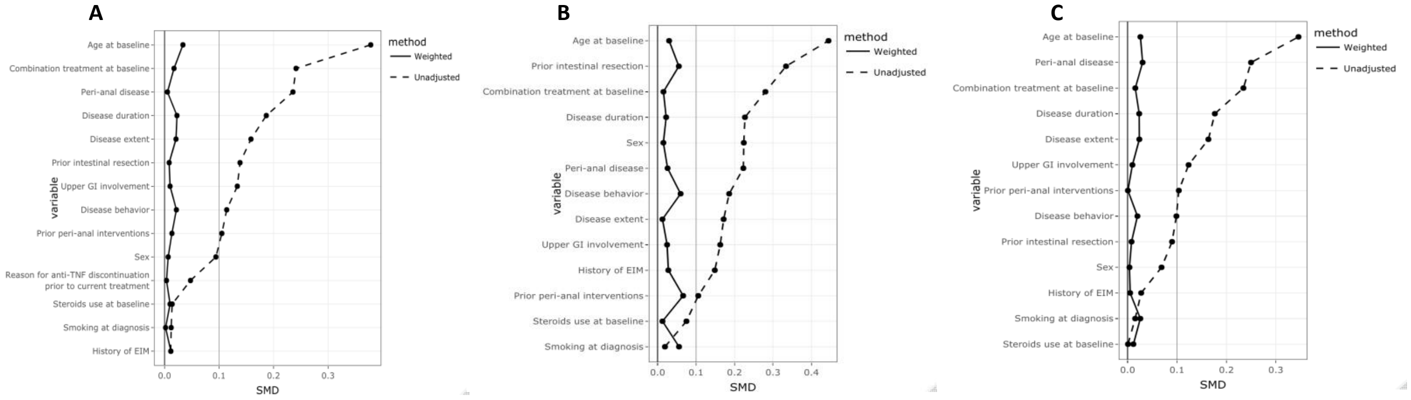

Supplemental Figure 6: SMD in baseline covariates between patients with CD who started a second-line biologic therapy after failure of anti-TNF. SMD of baseline confounding covariates that were included for estimating the propensity scores before (dotted lines) and after weighting (solid lines). Solid vertical line corresponds to SMD of 10% indicative of between group balance after weighting for comparisons between groups of patients starting relevant comparator treatments: (A) anti-TNF vs non-anti-TNF in all eligible patients; (B) anti-TNF vs non-anti-TNF in patients with PNR to first line anti-TNF; (C) anti-TNF vs non-anti-TNF in patients with NPNR to first line anti-TNF.

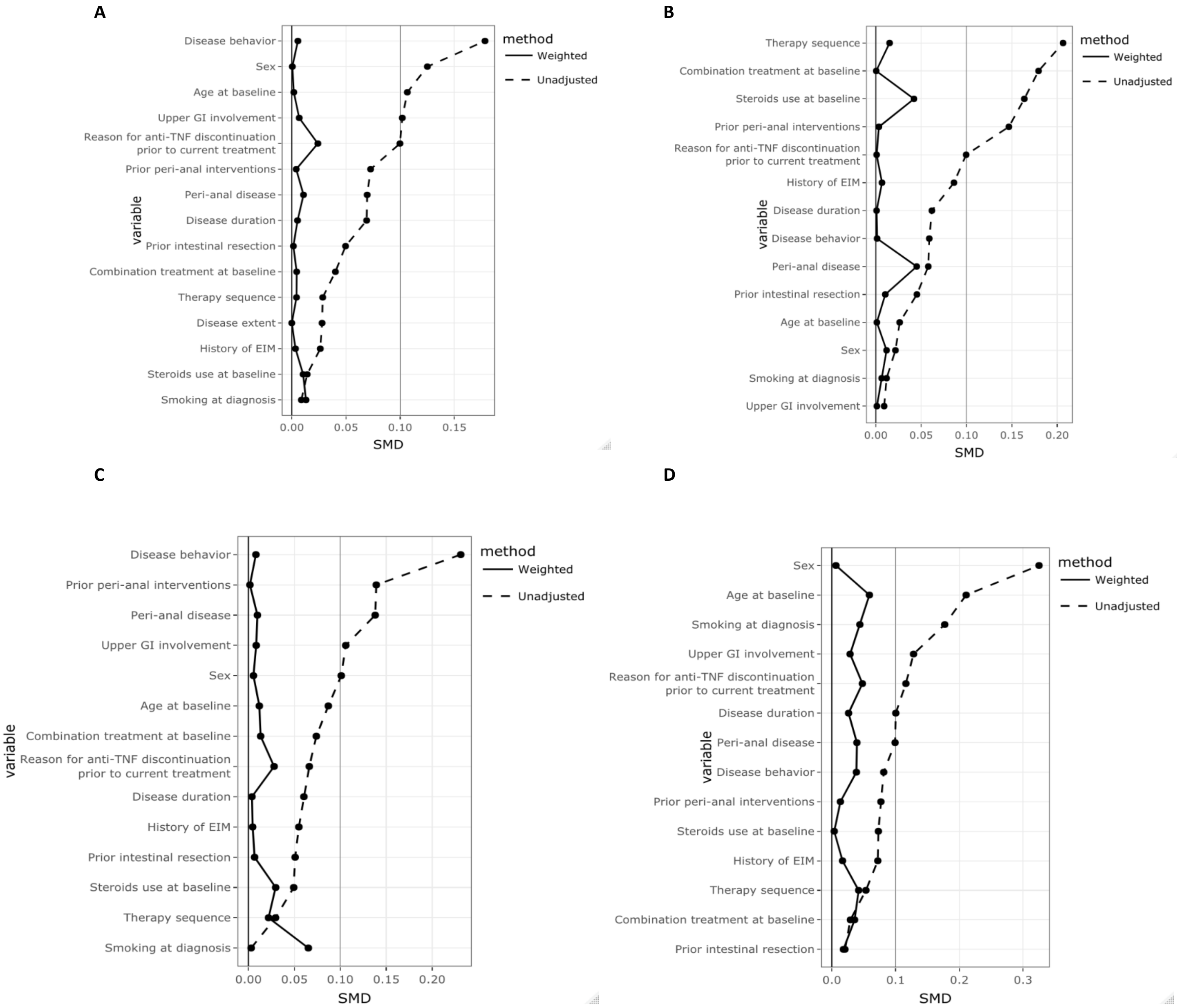

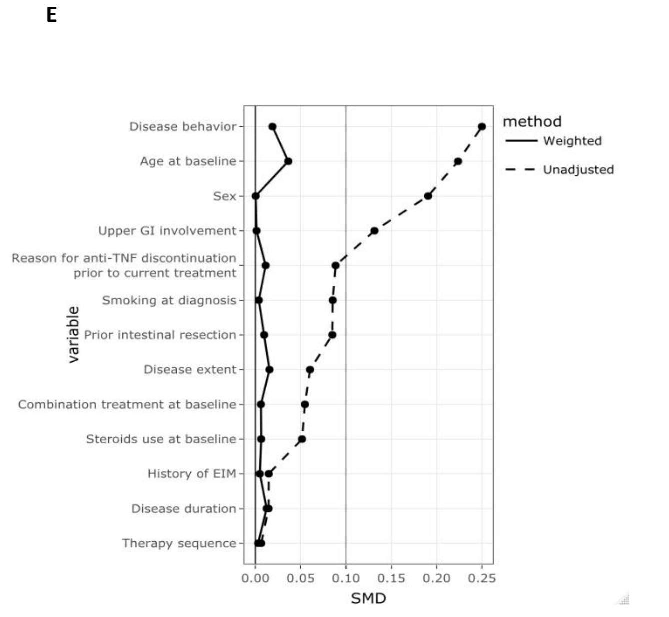

Supplemental Figure 7: SMD in baseline covariates between patients with CD who started UST vs VDZ therapy after failure of anti-TNF. SMD of baseline confounding covariates that were included for estimating the propensity scores before (dotted lines) and after weighting (solid lines). Solid vertical lines correspond to SMD of 10% indicative of between group balance after weighting for comparisons between groups of patients starting relevant comparator treatments: (A) UST vs VDZ after failure of anti-TNF in all eligible patients; (B) UST vs VDZ after failure of anti-TNF in patients with ileal CD; (C) UST vs VDZ after failure of anti-TNF in patients with ileocolonic CD; (D) UST vs VDZ after failure of anti-TNF in patients with colonic CD; (E) UST vs VDZ after failure of anti-TNF in patients with perianal CD.

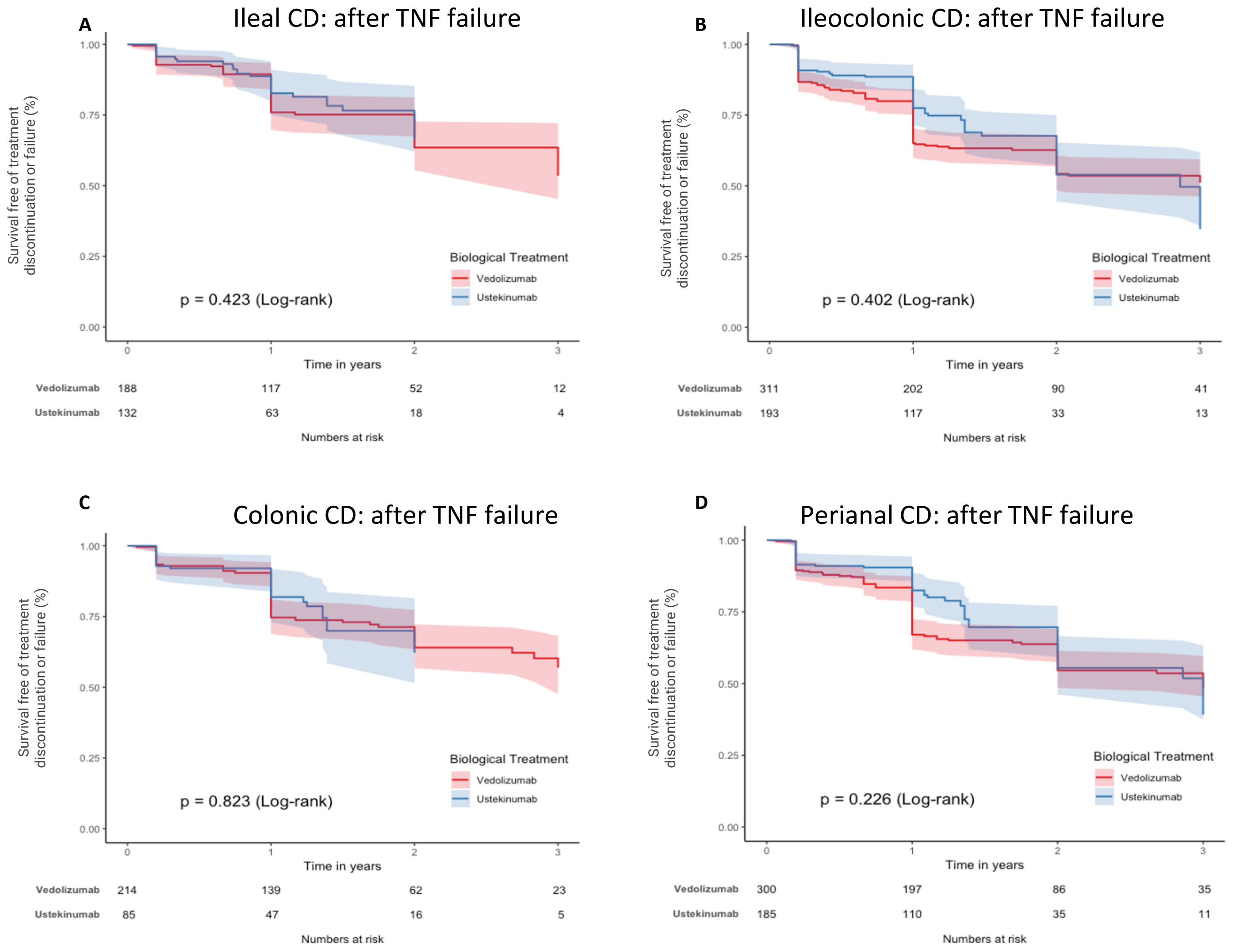

Supplemental Figures 8: Effectiveness of UST vs VDZ in CD after failure of anti-TNF. Kaplan-Meier plots depict survival free of treatment discontinuation or failure after IPTW adjustment for patients with CD treated for up to 3 years. **(A)** Use of VDZ (red) vs UST (blue) after failure of anti-TNF in patients with ileal CD; **(B)** Use of VDZ (red) vs UST (blue) after failure of anti-TNF in patients with ileocolonic CD; **(C)** Use of VDZ (red) vs UST (blue) after failure of anti-TNF in patients with colonic CD; **(D)** Use of VDZ (red) vs UST (blue) after failure of anti-TNF in patients with perianal CD. Log-rank p values as shown.

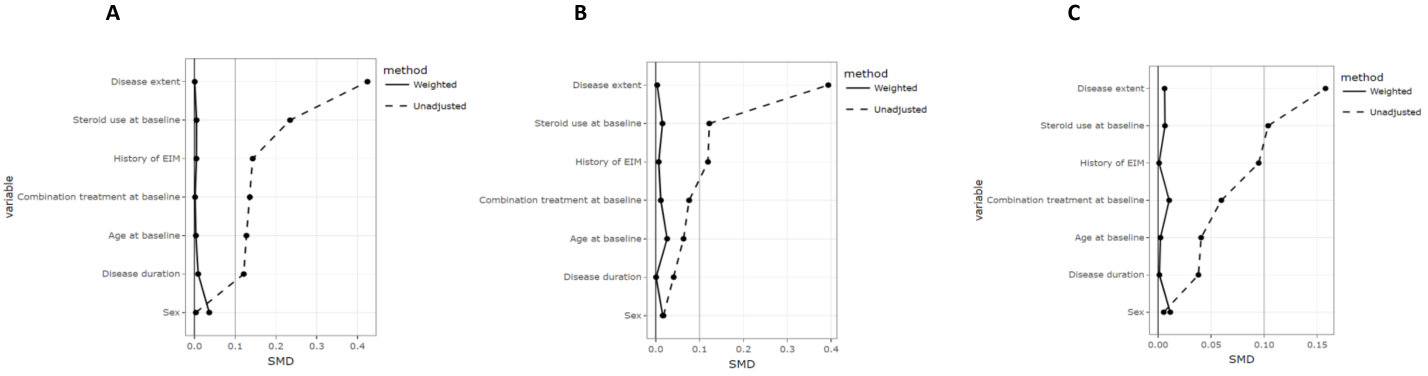

Supplemental Figure 9: SMD in baseline covariates between patients with UC who started VDZ in different sequences of therapy. SMD of baseline confounding covariates that were included for estimating the propensity scores before (dotted lines) and after weighting (solid lines). Solid vertical line corresponds to SMD of 10% indicative of between group balance after weighting for comparisons between groups of patients starting relevant comparator treatments: (A) 1st line VDZ vs 2nd line VDZ; (B) 1st line VDZ vs 3rd line VDZ; (C) 2nd line VDZ vs 3rd line VDZ.

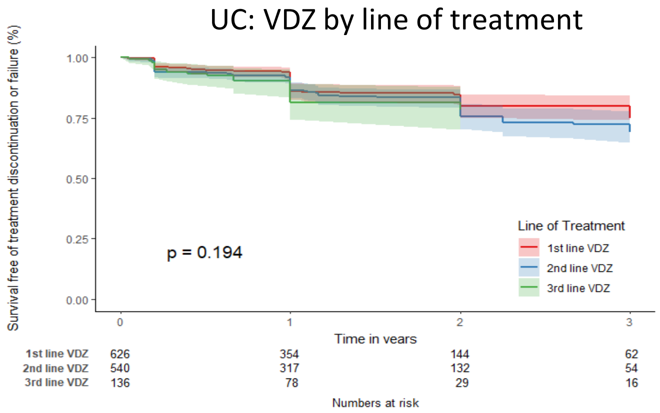

Supplemental Figure 10: Effectiveness of VDZ in UC according to line of therapy. Kaplan-Meier plots depict survival free of treatment discontinuation or failure after IPTW adjustment for patients with UC treated with VDZ for up to 3 years. Survival curves for patients receiving VDZ as first line (red), second line (blue) or third-line (green) therapy are shown. Log-rank p value as shown.

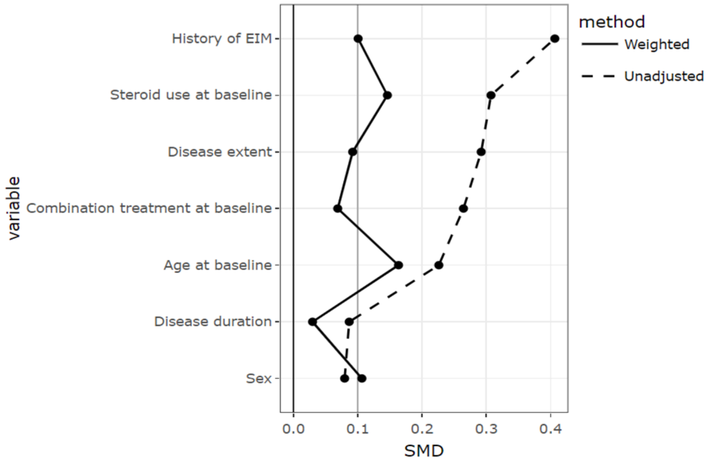

Supplemental Figure 11: SMD in baseline covariates between patients with UC who started anti-TNF in different sequences of therapy. SMD of baseline confounding covariates that were included for estimating the propensity scores before (dotted line) and after weighting (solid line). Solid vertical line corresponds to SMD of 10% indicative of between group balance after weighting for comparisons between groups of patients starting anti-TNF first line vs after VDZ failure.

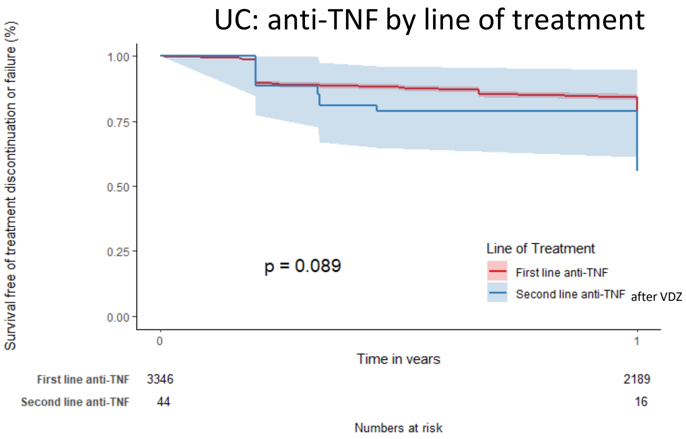

Supplemental Figure 12: Effectiveness of anti-TNF in UC as first-line therapy of after VDZ. Kaplan-Meier plots depict survival free of treatment discontinuation or failure after IPTW adjustment for patients with UC treated with anti-TNF for up to 1 year. Survival curves for patients receiving anti-TNF as first line (red) or after failure of VDZ (blue). Log-rank p value as shown.

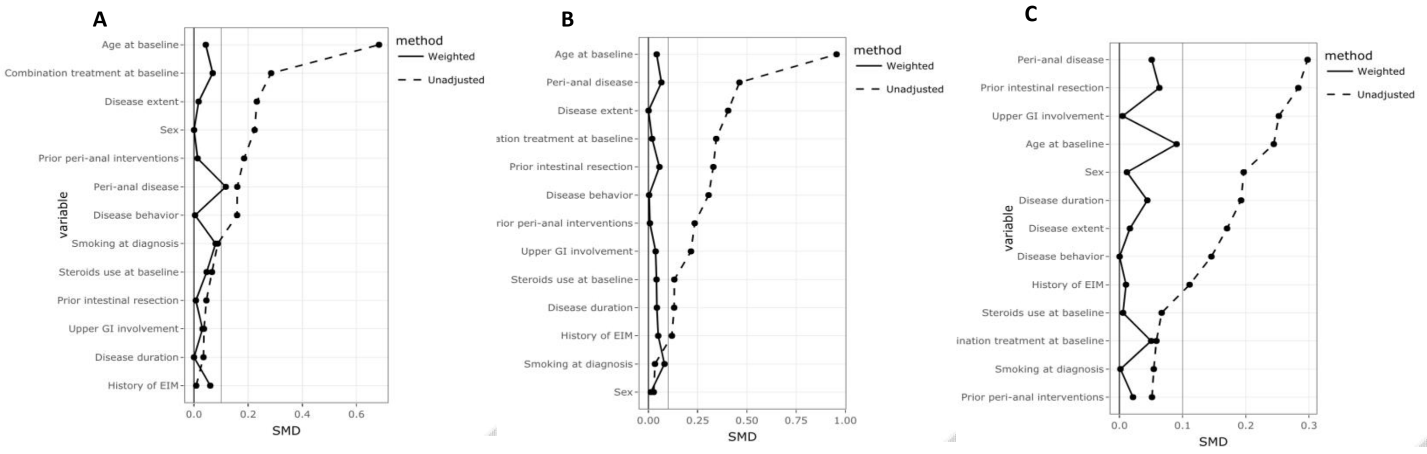

Supplemental Figure 13: SMD in baseline covariates between patients with CD who started VDZ in different sequences of therapy. SMD of baseline confounding covariates that were included for estimating the propensity scores before (dotted lines) and after weighting (solid lines). Solid vertical lines corresponds to SMD of 10% indicative of between group balance after weighting for comparisons between groups of patients starting relevant comparator treatments: (A) 1st line VDZ vs 2nd line VDZ; (B) 1st line VDZ vs 3rd line VDZ; (C) 2nd line VDZ vs 3rd line VDZ.

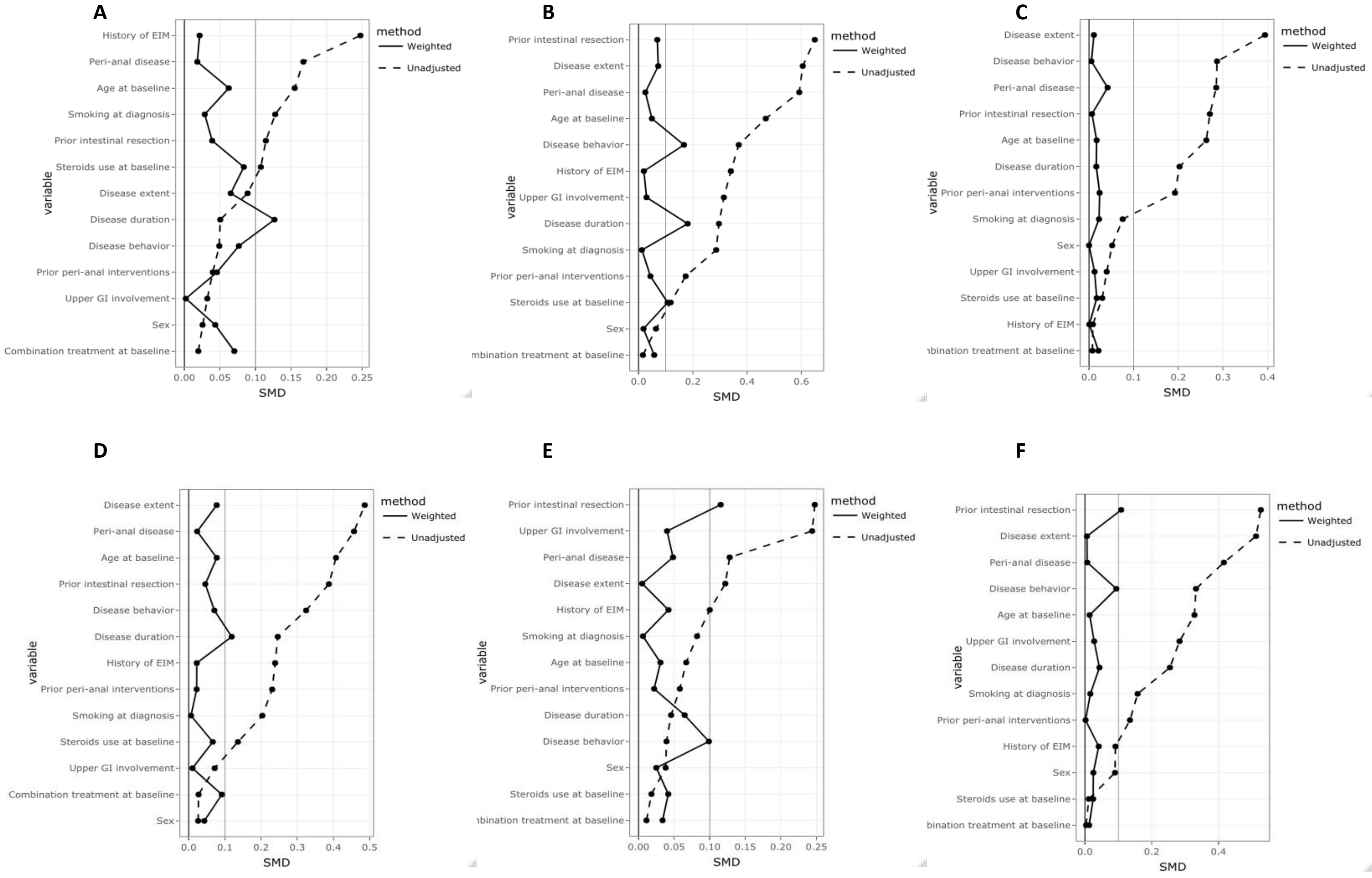

Supplemental figure 14: SMD in baseline covariates between patients with CD who started UST in different sequences of therapy. SMD of baseline confounding covariates that were included for estimating the propensity scores before (dotted lines) and after weighting (solid lines). Solid vertical lines corresponds to SMD of 10% indicative of between group balance after weighting for comparisons between groups of patients starting relevant comparator treatments: (A) 1st line UST vs 2nd line UST; (B) 1st line UST vs 4th line UST; (C) 2nd line UST vs 3rd line UST; (D) 1st line UST vs 3rd line UST; (E) 3rd line UST vs 4th line UST; (F) 2nd line UST vs 4rthline UST

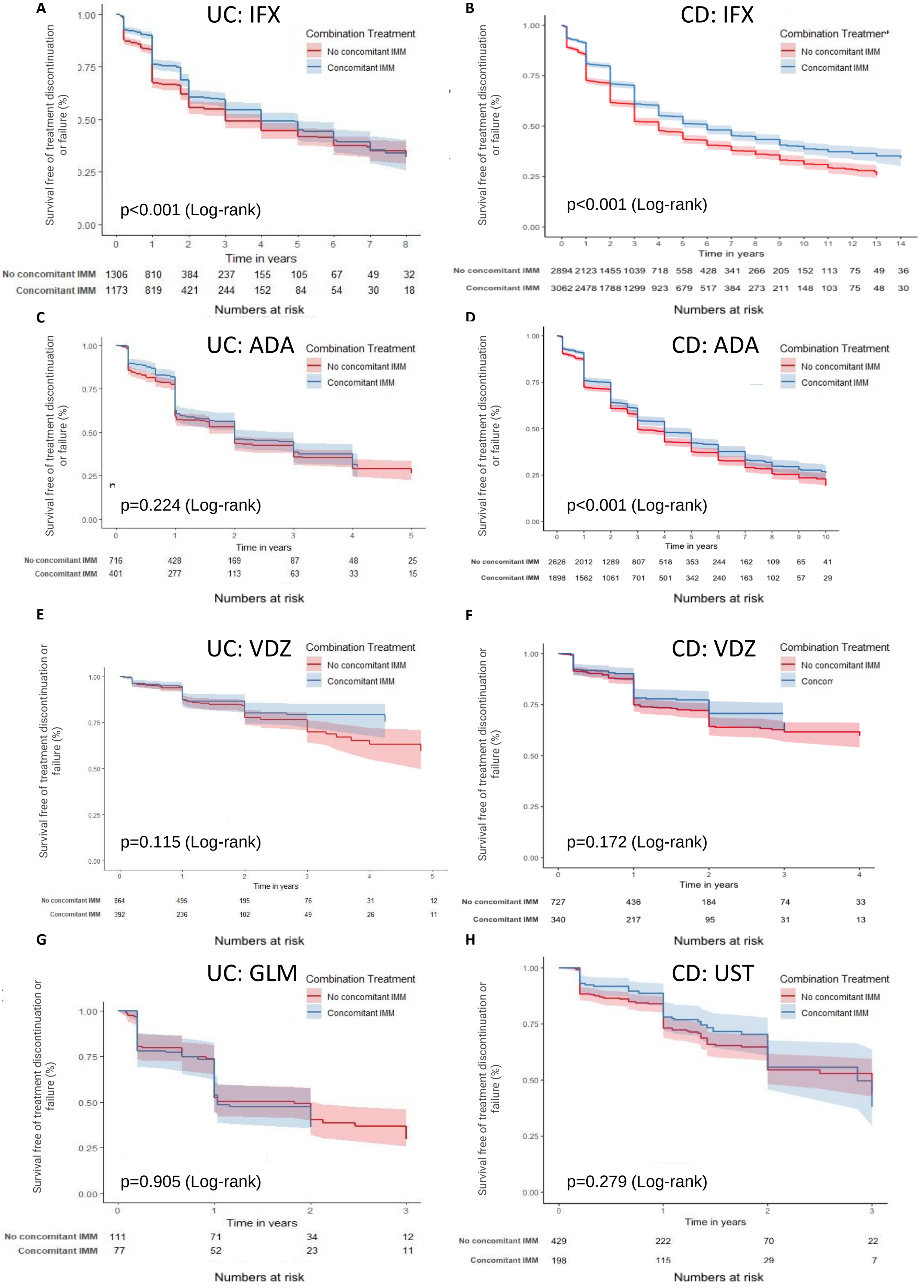

A
UC: IFX
Combination Treatment
No concomitant IMM
Concomitant IMM
Survival free of treatment discontinuation or failure (%)
p<0.001 (Log-rank)
Time in years
No concomitant IMM 1306 810 384 237 155 105 67 49 32
Concomitant IMM 1173 819 421 244 152 84 54 30 18
Numbers at risk
B
CD: IFX
Combination Treatment
No concomitant IMM
Concomitant IMM
Survival free of treatment discontinuation or failure (%)
p<0.001 (Log-rank)
Time in years
No concomitant IMM 2894 2123 1455 1039 718 558 428 341 266 205 152 113 75 49 36
Concomitant IMM 3062 2478 1788 1299 923 679 517 384 273 211 148 103 75 48 30
Numbers at risk
C
UC: ADA
Combination Treatment
No concomitant IMM
Concomitant IMM
Survival free of treatment discontinuation or failure (%)
p=0.224 (Log-rank)
Time in years
No concomitant IMM 716 428 169 87 48 25
Concomitant IMM 401 277 113 63 33 15
Numbers at risk
D
CD: ADA
Combination Treatment
No concomitant IMM
Concomitant IMM
Survival free of treatment discontinuation or failure (%)
p<0.001 (Log-rank)
Time in years
No concomitant IMM 2626 2012 1289 807 518 353 244 162 109 65 41
Concomitant IMM 1898 1562 1061 701 501 342 240 163 102 57 29
Numbers at risk
E
UC: VDZ
Combination Treatment
No concomitant IMM
Concomitant IMM
Survival free of treatment discontinuation or failure (%)
p=0.115 (Log-rank)
Time in years
No concomitant IMM 864 495 195 76 31 12
Concomitant IMM 392 236 102 49 26 11
Numbers at risk
F
CD: VDZ
Combination Treatment
No concomitant IMM
Concom
Survival free of treatment discontinuation or failure (%)
p=0.172 (Log-rank)
Time in years
No concomitant IMM 727 436 184 74 33
Concomitant IMM 340 217 95 31 13
Numbers at risk
G
UC: GLM
Combination Treatment
No concomitant IMM
Concomitant IMM
Survival free of treatment discontinuation or failure (%)
p=0.905 (Log-rank)
Time in years
No concomitant IMM 111 71 34 12
Concomitant IMM 77 52 23 11
Numbers at risk
H
CD: UST
Combination Treatment
No concomitant IMM
Concomitant IMM
Survival free of treatment discontinuation or failure (%)
p=0.279 (Log-rank)
Time in years
No concomitant IMM 429 222 70 22
Concomitant IMM 198 115 29 7
Numbers at risk

I

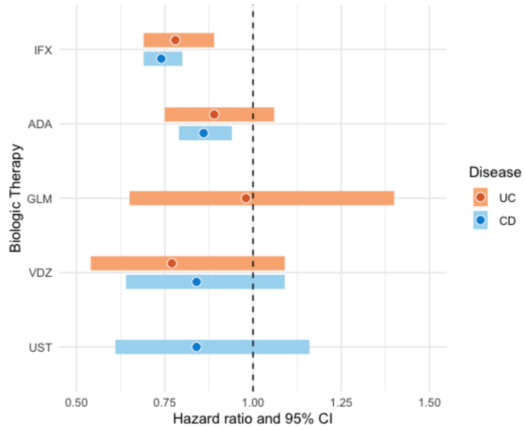

Supplemental figure 15: Effectiveness of biologic therapy in patients with UC and CD according to concomitant use of immunomodulators. Kaplan-Meier plots depict survival free of treatment discontinuation or failure after IPTW adjustment for patients with **(A)** UC and IFX; **(B)** CD and IFX; **(C)** UC and ADA; **(D)** CD and ADA; **(E)** UC and VDZ; **(F)** CD and VDZ; **(G)** UC and GLM; **(H)** CD and UST; **(I)** summary plot depicting HRs and 95% CI for treatment discontinuation or failure with concomitant use of immunomodulator for each biologic therapy in UC and CD
